# Differences by origin in methylome suggest eco‐phenotypes in the kelp *Saccharina latissima*

**DOI:** 10.1111/eva.13382

**Published:** 2022-05-11

**Authors:** Lydia Scheschonk, Kai Bischof, Martina Elisabeth Luise Kopp, Alexander Jueterbock

**Affiliations:** ^1^ 9168 Marine Botany & MARUM University of Bremen Bremen Germany; ^2^ 1786 Genomics and Ecology Research Groups Faculty of Biosciences and Aquaculture Nord University Bodø Norway; ^3^ 1786 Algal and Microbial Biotechnology Division Faculty of Biosciences and Aquaculture Nord University Bodø Norway

**Keywords:** aquaculture, DNA methylation, eco‐phenotype, epigenetics, kelp, priming, temperature adaptation

## Abstract

Most kelp species are of high ecological and economic importance worldwide, but are highly susceptible to rising ocean temperatures due to their sessile lifestyle. Due to interference with reproduction, development and growth, natural kelp forests have vanished in multiple regions after extreme summer heat waves. Furthermore, increasing temperatures are likely to decrease biomass production and, thus, reduce production security of farmed kelp. Epigenetic variation, and cytosine methylation as a heritable epigenetic trait, is a rapid means of acclimation and adaptation to environmental conditions, including temperature. While the first methylome of brown macroalgae has been recently described in the kelp *Saccharina japonica*, its functional relevance and contribution to environmental acclimation is currently unknown. The main objective of our study was to identify the importance of the methylome in the congener kelp species *Saccharina latissima* for temperature acclimation. Our study is the first to compare DNA methylation in kelp between wild populations of different latitudinal origin, and the first to investigate the effect of cultivation and rearing temperature on genome‐wide cytosine methylation. Origin appears to determine many traits in kelp, but it is unknown to what extent the effects of thermal acclimation may be overruled by lab‐related acclimation. Our results suggest that seaweed hatchery conditions have strong effects on the methylome and, thus, putatively on the epigenetically controlled characteristics of young kelp sporophytes. However, culture origin could best explain epigenetic differences in our samples suggesting that epigenetic mechanisms contribute to local adaptation of eco‐phenotypes. Our study is a first step to understand whether DNA methylation marks (via their effect on gene regulation) may be used as biological regulators to enhance production security and kelp restoration success under rising temperatures, and highlights the importance to match hatchery conditions to origin.

## 1 INTRODUCTION

Marine macroalgae are the foundation of marine rocky shore ecosystems, provide nursery grounds for commercially important fishes and promote bio‐remediation and carbon sequestration (Bartsch et al., 2008; Duffy et al., 2019; Teagle et al., 2017). Traditionally cultivated in Asia, macroalgal cultivation is gaining momentum in other regions such as Europe and America as an environmentally sustainable new blue bio‐economy (Cai et al., 2021). In 2019, ~30% of the global production of marine aquaculture commodities (120 million t) could be accounted for by marine macroalgae, worth US$ 14.9 billion (Cai et al., 2021).

The boreal‐temperate brown alga *Saccharina latissima* (sugar kelp) is of high ecological and economic value (Bartsch et al., 2008; Cai et al., 2021). In Europe, populations inhabit rocky shores from the north of Portugal up to Spitsbergen (Norway). As in all kelp, the life cycle of *S*. *latissima* entails a microscopic gametophyte stage (1n) divided into male and female individuals, and a macroscopic sporophyte stage (2n). Sporophytes are the stage visible as kelp forest, and the habitus typically comprises a holdfast, stipe and blade. The strongest factors determining range limits in kelp are the respective thermal limits of gameto‐ and sporophyte stages (Lämke & Bäurle, 2017). Due to increasing (summer) water temperatures caused by climate change, the species’ distributional range is shifting northwards (Bringloe et al., 2020; Krause‐Jensen et al., 2020). Even where rising temperatures remain below lethal limits, they frequently become high enough to compromise growth (Gerard, 1997), and are expected to increase disease outbreaks and bio‐fouling (Harley et al., 2012).

In general, organisms can respond to environmental change in four ways: move, adapt, cope or die (Gienapp et al., 2008). As sessile organisms with short dispersal phases, kelps are particularly vulnerable to ocean warming. In consequence, their survival largely depends on their potential to acclimate and adapt. Recent studies in the budding research field of ecological epigenetics suggest that the DNA sequence by itself is not the single most important component of adaptation and population persistence (Richards & Pigliucci, 2020). Research efforts aiming to understand epigenetic variation and differentiation are growing strong in terrestrial plants (Richards et al., 2017). The studies provide increasing evidence for the evolutionary potential in this hitherto overlooked level of intraspecific variation. Stress‐responsive shifts in the epigenome mediate phenotypic changes that can allow for genomic changes at a later stage (Richards et al., 2012, 2017). Moreover, epigenomic responses to the environment can be stable over generations (Holeski et al., 2012). Such epigenetic inheritance (Jablonka & Raz, 2009) might best be described as ‘transgenerational priming’: a plants’ ability to create a stress memory that is passed on from the parent generation via a heritable epigenetic mechanism (see Jueterbock et al., 2021). Several epigenetic mechanisms are known: histone modifications, noncoding RNAs and cytosine methylation (Bossdorf et al., 2008). Among these mechanisms, the first two facilitate rather ‘instant’ modulations that are generally not transferred across generations. Cytosine methylation, however, can be heritable across several generations (Lämke & Bäurle, 2017) and, thus, is a likely candidate for rapid non‐genetic but heritable local adaptation. In marine primary producers, epigenome research is lagging behind. In brown macroalgae (*Phaeophyceae*), cytosine DNA methylation is poorly understood, and appears to be present in only some species. In the brown algal model organism *Ectocarpus siliculosus*, DNA methylation is presumed to be negligible (<0.035%, Cock et al., 2010). In contrast, it apparently is present in the kelp *Saccharina japonica* at a level of 1.4% (Fan et al., 2020), but is still low compared to terrestrial plants. Plants generally show 4.8%–42.2% genome‐wide cytosine methylation (Alonso et al., 2019), with 2%–86% in gene bodies across *Viridiplantae* (Bewick et al., 2017). Despite the lower level, cytosine methylation appears to be involved in metabolic and life‐cycle regulation in the kelp *S*. *japonica* (Fan et al., 2020). Young sporophytes of *S*. *latissima* in culture can be assigned to different origins by their distinct habitus (Heinrich et al., 2016; Monteiro, 2020), hinting at phenotypic plasticity and/or local adaptations. These presumably heritable traits, specific to each origin, might result in different responses between origins to cultivation factors. Of these factors, temperature is likely to be one of the most important drivers of plasticity and adaptation, and, ultimately, the main factor driving geographical distribution (Lämke & Bäurle, 2017). Our study aimed at determining the influence of temperature, origin and rearing condition (field vs. lab grown) on the methylome of kelp. We tested the following hypotheses:

1. The methylome of the kelp *S*. *latissima* resembles the one of *S*. *japonica*, in contrast to being absent as in the brown algal evo‐devo model *E*. *siliculosus* (Cock et al., 2010).
2. Eco‐phenotypes (definition: see Discussion ‘Eco‐phenotypes’, or Glossary), adapted to local conditions from clearly distinct latitudes (= origins), show contrasting methylation patterns despite being cultivated under equal conditions. This would indicate that kelp can epigenetically ‘memorize’ the ambient conditions experienced by previous generations through transgenerational stability of origin‐specific methylation marks (= transgenerational priming).
3. The species’ affinity to cold environments, indicated by recent climate change‐induced range shifts towards high latitudes, should be mirrored in methylomes from high‐latitude origins presenting methylation patterns/levels indicating low stress: Presuming that the genome‐wide methylation is positively related to the amount of stress (Wibowo et al., 2016), we hypothesize that methylation should be more abundant in individuals from the lower‐latitude origin that repeatedly experienced the abiotic factor (‘temperature‘) at stress level (‘heat’).

## 2 MATERIALS AND METHODS

### 2.1 Lab cultures

Clonal gametophyte cultures of *Saccharina latissima* (Linnaeus) C.E. Lane, C. Mayes, Druehl & G.W. Saunders from Helgoland, German Bight (Nordstrand, 54°11′18.9″N 7°54′14.1″E; HG1, HG2, HG3 and HG4 in culture since 2014), and Spitsbergen, Svalbard (Hansneset, Kongsfjorden, 78°59′26.0″N 11°58′42.3″E; SG1, SG2, SG3 and SG4 in culture since 2015) were mono‐parentally fertilized. Gametophytes had been kept under identical conditions. As all gametophytes within a respective culture (e.g. HG1) were grown as clones stemming from the same zoospore (which is a single cell), all gametophytes within this culture (e.g. HG1) can be presumed to be true clones. In the resulting sporophyte cultures (Helgoland: HG1♀ X HG1♂ = H1, HG2♀ X HG2♂ = H2, HG3♀ X HG3♂ = H3, HG4♀ X HG4♂ = H4; Spitsbergen: SG1♀ X SG1♂ = S1, S2♀ X S2♂ = S2, S3♀ X S3♂ = S3, S34 X S4♂ = S4; see Figure S1), all sporophytes within a culture (e.g. H1) were twins (but not clones anymore), as meiotic processes (such as chromosome recombination) occur. The process of uniparental fertilization, called selfing, can lead to heterozygote deficiency and inbreeding. Inbreeding, estimated by the inbreeding coefficient F_IS_, has been found to be significant in wild populations of *S*. *latissima* (F_IS_ between 0.081 and 0.187 (Skagerrak/ Kattegat/Baltic sea; Møller Nielsen et al., 2016)). Thus, while we cannot fully exclude the possibility, we presume that our comparison between lab and field samples is not significantly impaired by a possible high level of inbreeding in our lab samples.

Sporophyte cultures were initially kept for ≥6 weeks in petri dishes in autoclaved natural seawater (Salinity 34) that was Provasoli enriched (PES) with half the concentration used for grown sporophytes (= ½ PES). As soon as fertilization was initiated, the four cultures per origin (S1–S4, H1–H4; technical replicates) were kept at each of the three temperatures (5, 10, and 15°C) under white light at 15 µmol/m^2^/s (18:6 h light:dark). PES in petri dishes was changed every ~10 days. During each water change, only the largest individuals were maintained in culture, while smaller plants were discarded. Growth rates between the temperatures, but not origins, differed strongly. When the sporophytes in a petri dish reached a length of about 2 cm, they were transferred to aerated 1‐l Schott bottles (with sterile ½ PES; five plants per bottle). At a length of about 5–7 cm, ½ PES was changed to full PES, exchanged at least every 7 days. At a length of 12cm, sporophytes were moved to aerated 3‐l beakers with sterile full PES that was exchanged at least once a week.

From each culture, sporophytes of corresponding size (>4 per beaker) were frozen at −80°C. After all samples had been obtained, they were placed into cellulose bags—the bags were covered in silica gel—to dry; only sporophytes with a fresh weight exceeding 120 mg (without stipe) were taken for DNA extraction. One dried sporophyte per beaker was randomly chosen as sample for extraction. In three beakers, no sporophytes had developed successfully, so in total 21 instead of 24 lab samples could be sequenced.

### 2.2 Field samples

Adult sporophytes were sampled from Helgoland, German Bight (54°11′18.9″N 7°54′14.1″E), during the last week of May 2019 (*n* = 10, ~10°C at 10 m depth at time of sampling; annual ~max/~min: ~19°C/~5°C; see Figure S2), and from Spitsbergen, Svalbard (78°59′26.0″N 11°58′42.3″E), during the last week of June 2019 (*n* = 10, ~5°C at 10 m depth at time of sampling; annual ~max/~min: ~7.5°C/~−1.4°C; see Figure S3), at the same locations from which the gametophytes for the lab‐grown sporophytes originated. As seasons, and with this, circannual processes, are shifted in timing in the high Arctic relative to temperate regions, we presume the difference in sampling time to be negligible.

The same scuba diver sampled on Helgoland and Spitsbergen. At each site, 10 sporophytes were retrieved from corresponding depths. From each sporophyte, four discs (ø = 3 cm) were cut from the fronds, omitting the meristematic region, ‘midsection’ and reproductive tissues. All tissue samples (lab and field) were dried in cellulose bags in silica. Prior to DNA extraction, all predried samples (lab and field) were placed in an oven and dried overnight at ~45°C. In total, 21 samples from lab culture and 20 samples from the wild were analysed.

### 2.3 DNA extraction

Dried tissue samples were powdered (Fastprep with porcelain beads, 60 s @ 30 Hz, vortex, 20 s @ 30 Hz) prior to extraction. DNA was extracted with E.Z.N.A^®^ HP Plant DNA Mini kit (D2485, Omega Bio‐tek Inc.) from 10 mg powdered dry tissue following the protocol for dried specimens (pp. 5–8 in manual v5.0 from July 2019) with application of RNase A and 2‐mercaptoethanol, the column equilibration protocol and 5 min of incubation after the application of elution buffer. Extraction was followed by a clean‐up and concentration procedure using Zymo Research Clean & Concentrator™ 5 µg (Zymoresearch, manual Ver.1.3.1) with the following adjustment: with two rounds of elution in ≥30 µl ultrapure H_2_O. DNA extraction yield was checked with a Nanodrop (ND‐1000 UV‐Vis Spectrometer and Agilent 2100 Bioanalyzer, Agilent Technologies). Extracted DNA was lyophilized (Alpha 1–4 LO plus, Martin Christ Gefriertrockungsanlagen GMBH) for transport, and rehydrated with nuclease‐free water prior to library preparation.

### 2.4 MethylRAD sequencing

Sequencing libraries were prepared from 100 ng DNA according to the MethylRAD protocol by Wang et al. (2015). MethylRAD utilizes the methylation‐dependent restriction enzyme FspEI that targets fully methylated CCGG and CCHGG motifs, hence capturing methylation in CG and CHG sequence contexts. MethylRAD has the potential to reveal genome‐wide DNA methylation patterns that are consistent with those generated from Whole Genome Bisulfite Sequencing (WGBS, Wang et al., 2015). Cytosine DNA methylation in plants usually occurs in CG, CHG and CHH sequence contexts (H = any base but G; Bewick et al., 2017). CHH methylation is the least stable across mitotic cell divisions (Boquete et al., 2021), while CG is the most stable during mitotic and meiotic cell division (Schmitz et al., 2019). Genes are usually methylated in the CG context only, while all three contexts are found to methylate transposons, with CHH being the most abundant here, but the only nonheritable (Boquete et al., 2021; Dubin et al., 2015; Schmitz et al., 2019). Thus, omission of CHH methylation in our study provides focus on the methylation contexts that have highest potential for long‐term, transgenerational acclimation and nongenetic adaptation.

As compared with the original MethylRAD protocol (Wang et al., 2015), we applied the following adjustments. First, sense and antisense oligos of adapters A1 and A2 (sequence: see table S5 in Wang et al., 2015) were annealed in 10 μl containing 7 μl nuclease‐free H_2_O, 1 μl of each oligo (100 μM stock concentration, Eurofins) and 1 μl 10× annealing buffer (containing 10 mM Tris HCl (pH 8, ThermoFisher), 50 mM NaCl (ThermoFisher) and 1 mM EDTA (pH 8, ThermoFisher)).

Library preparation began with digestion of 100 ng cleaned genomic DNA (in 12.2 μl nuclease free water) in a total volume of 15 μl, containing 0.8 μl FspEI (5 U/μl, NEB), 1.5 μl 10× CutSmart Buffer (NEB) and 0.5 μl 30× Enzyme Activator Solution (NEB), at 37°C for 4 h.

Digestion was verified on a TapeStation 2200 with a D1000 ScreenTape. After tape station verification, adapters were ligated to the digested fragments overnight (~12 h) at 4°C in 20 μl with 10 μl digested solution and 10 μl ligation master mix, containing 0.26 µl of each of two adaptors (10 µM stock), 1.3 µl ATP (10 mM, NEB), 1040 U of T4 DNA ligase (NEB), 2.6 µl 10× T4 ligase buffer (with 10 mM ATP, NEB) and 5.98 µl nuclease‐free water.

Ligation products were amplified in 20‐μl reactions of 7 μl ligated DNA and 13 μl master mix containing 0.2 μM of each primer (P1 and P2, 10 μM stock; sequence: see Wang et al., 2015), 0.6 μl 10 mM dNTP, 4 μl 5× Phusion HF buffer (NEB) and 0.2 μl Phusion high‐fidelity DNA polymerase (2 U/μl, NEB). PCR was conducted using a Veriti 96‐Well Thermal Cycler (Applied Biosystems, Life Technologies) with 16 cycles at 98°C for 5 s, 60°C for 20 s, 72°C for 10 s and a final extension of 5 min at 72°C. The target band (approx. 100 bp) was extracted from a 2% E‐Gel (Thermo Fisher). The concentration of the PCR product of about 100 bp was verified with a Qubit dsDNA HS Assay kit (Life Technologies) using a Qubit 4.0 Fluorometer (Life Technologies).

Sample barcodes were introduced by means of PCR. Each 20 μl PCR reaction contained an individual barcode (index primer), 12 μl of gel‐extracted PCR product and 8 µl master mix. The master mix contained 2.4 µl nuclease‐free H2O, 4 µl 5× Phusion HF Buffer, 0.4 µl 10 µM primer p3 (sequence: see Wang et al., 2015), 0.6 μl 10 mM dNTP and 0.2 µl Phusion high‐fidelity DNA polymerase (2 U/µl). PCR was conducted with the same PCR cycling program outlined above; the final PCR product was at ~156 bp. After the final PCR, PCR products were purified using AMPURE XP beads (Beckman Coulter) using a 1.8: 1 volume ratio of beads to product, and a final elution in 22 μl EB buffer (Qiagen). The purified fragments of the 41 samples were sequenced using the NEBNext Ultra II DNA Library Prep kit on an Illumina NextSeq 500 (1 × 75 bp) preparing two NextSeq 500/550 Mid output kit v 2.5 (NEB): The 21 experimental/lab samples and the 20 field samples were sequenced on two separate flow cells due to logistical reasons.

### 2.5 Bioinformatics

#### 2.5.1 Raw read clean‐up

The sequences of all four lanes were pooled per sample, and quality trimmed with TrimGalore! v 0.4.1 (https://www.bioinformatics.babraham.ac.uk/projects/trim_galore/) with elimination of bases with Phred score <20 (‐‐quality 20), removal of the adapter sequence (‐‐stringency 3) and removal of terminal 2bp (both ends) to eliminate artefacts that might have arisen at the ligation position (‐‐clip_R1 2\‐‐three_prime_clip_R1 2). Before (491,169,744 RawReads, Table S1) and after quality trimming (476,161,873 TrimmedReads, Table S1), we quality checked the reads for fragment (over‐) representation, base AT CG content bias and actual fragment length/length of reads, using FastQc v0.11.8.

#### 2.5.2 In silico digestion

As the genome of *Saccharina latissima* remains unpublished as of now, we mapped the high‐quality reads to the genome of the closely related species *Saccharina japonica* (Fan et al., 2020; v6.2 from ORCAE (Sterck et al., 2012)). The 476,161,873 trimmed reads (All TrimmedReads, Table S1) were mapped with the SOAP aligner (v1.11, Li et al., 2008) to 548,536,073 *in silico* digested MethylRAD tags (1,793,114 with CCGG recognition sites, 527,987 CCTGG, 496,183 CCTGT and 527,729 CCAGG) that we had extracted from the *S*. *japonica* genome with a custom python script *InSilicoTypeIIbDigestion_corrected*.*py* (https://gist.github.com/alj1983/a1bcb950013d8ffac9a7fb9ceb70b1c0). For mapping, we used the following specifications: two mismatches allowed *(*‐*v 2*), sanger quality +33 *(*‐*z!*), filter out reads containing >1N *(*‐*f 1*), a seed size of 8 (‐*s 8*), amounting to 10,938,502 reads (All MappedReads, Table S1). The coverage (amount of reads that mapped here) of each methylated site in each sample was derived from uniquely mapped reads (4,016,216 All UniquelyMappedReads, Table S1) using htseq count (v 0.7.2, biohawk v20110810, samtools v1.9 (using htslib 1.9)). Methylated sites were retained for further analysis only when supported with a coverage ≥3 (111,992 sites; number of rows in Table S2). For each sample, raw counts were normalized to reads per million by dividing reads per site through the total number of reads per sample library, times 1 million.

#### 2.5.3 Annotation

We annotated the methylation sites for genomic regions and gene IDs based on the *S*. *japonica* annotation gff3 file from the ORCAE database (version uploaded on May 26 2020, https://bioinformatics.psb.ugent.be/gdb/Saccharina/) with the R package ‘GenomicRanges’ v 1.44.0 (Lawrence et al., 2013). As genomic regions we defined genes (exons and introns), promoter regions (2000 bp upstream to 200 bp downstream the start codon), transcription start sites (300 bp upstream to 50 bp downstream the start codon), 10 kbp up‐ and downstream regions flanking the gene body, repeats in any genomic region and intergenic regions not included in the gene body or the 10 kbp flanking regions.

#### 2.5.4 Check for microbial contamination

Since only a minor fraction of the sequenced reads (~1% ±1.0 SD; see Table S1, Column F) mapped back to the reference genome of *S*. *japonica*, we checked for microbial contamination.

First, we assembled a draft reference genome for one randomly chosen *S*. *latissima* lab sample. For this sample, we prepared a genome library with NEBNext Ultra II DNA Library Prep kit for Illumina with the NextSeq 500/550 Mid output kit v 2.5 (NEB) and sequenced it on the Illumina NextSeq (2 × 150bp). Raw reads (14.15 million) were quality checked with FastQC v0.11.5 to control for aberrant read base content, length distribution, duplication and over‐ representation. We used TrimGalore! v0.6.0 to remove adapter sequences with a stringency of 3 bp overlap, reads <20 bp and low‐quality bases with a Phred score Q < 20 (99% base call accuracy). The high‐quality reads (14.12 million) were assembled with Minia v.3 (Chikhi & Rizk, 2013) using a k‐mer of length 25. A 25 kmer had been identified as optimal (compared with 77, 99 and 127 bp) by kmergenie v. 1.7051 and, based on Quast (Gurevich et al., 2013), resulted in the largest number of contigs (2611) above 1000 bp.

The first approach to estimate the proportion of contamination with DNA of the nontarget species was to quantify the ratio of the high‐quality reads that map back to the reference genome of *Saccharina japonica* (Fan et al., 2020) using BWA v0.7.12‐r1039 (Li, 2013, https://arxiv.org/abs/1303.3997) with standard settings.

The second approach to quantify and qualify DNA contamination was to taxonomically label the de novo assembled genomic contigs with BlobTools (Laetsch et al., 2017). We followed the BlobTools de novo workflow by creating a BlobTools database and plotting the taxonomic annotation based on (1) the 1,486,627 assembled contigs, (2) a Blast (Altschul et al., 1990) report provided by a blastn query against the nt database (blastn ‐db BLAST/nt ‐query KMER25.contigs.fa ‐outfmt ‘6 qseqid staxids bitscore std’ ‐max_target_seqs 10 ‐max_hsps 1 ‐evalue 1e‐25 >BlastOutputForBlobTools.txt) and (3) a bam file of mapped high‐quality reads against the assembled contigs using BWA v0.7.12‐r1039 (see above) with standard settings.

### 2.6 Statistics

During statistical analyses, ‘lab’ entailed all samples from the experimental setup regardless of temperature or origin. ‘field’ entailed all field samples regardless of origin. Only uniquely mapped reads with sites covered ≥3 (see ‘in‐silico digestion’) were taken into statistical analyses.

### 2.7 Principal component analysis (PCA) and hierarchical clustering

Overall methylome differentiation among samples was characterized with the principal components analysis (PCA) in the R package ‘FACTOMINER’ (Lê et al., 2008; setting scale.unit = FALSE not to scale the expression values to unit variance). Input values were raw data standardized to reads per million (RPM), see Table S3.

### 2.8 DNA methylation levels

We tested for significant differences in DNA methylation levels (reads per million), and the number of methylated sites between cultivation temperatures with Wilcoxon rank sum tests followed by correcting the *p*‐values for multiple pairwise comparison (*p*adj > 0.05) using the Benjamini–Hochberg method (Benjamini & Hochberg, 1995) with the R package ‘rstatix’ v0.6.0 (Kassambara, 2020).

### 2.9 Differential methylation

**FIGURE 1:**
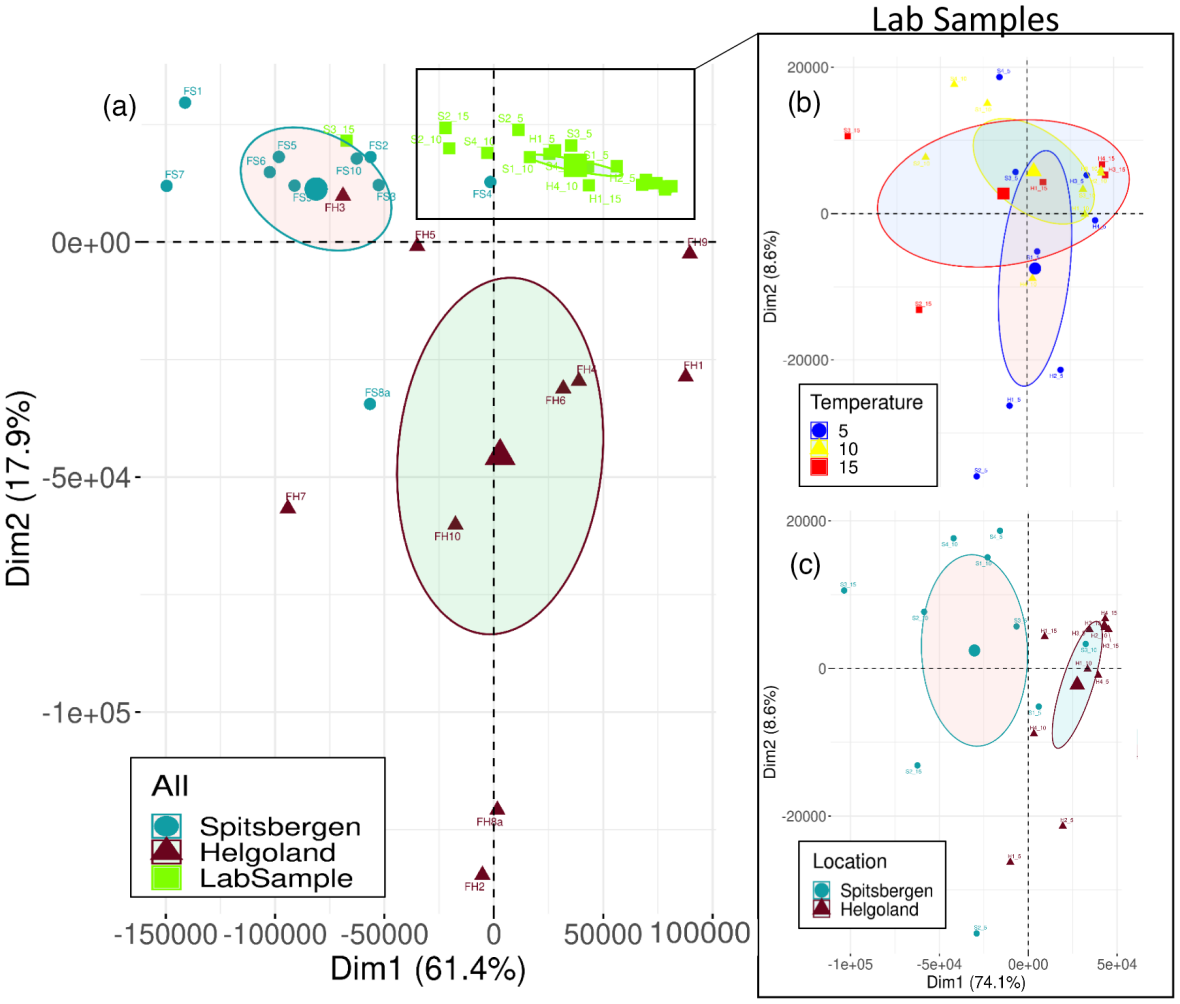
Principal component analyses (PCA) for (a) all samples of both origins (Spitsbergen and Helgoland) and rearing conditions (lab and field), and for the lab samples (‘zoomed in’ from a) for (b) ‘temperature’ and (c) ‘location’, with 95% confidence ellipses. PCA calculated with the R package ‘FACTOMINER’ (Lê et al., 2008) showed a definite influence of cultivation on the methylome, followed by origin, especially in the field samples. No significant differences could be found between temperatures in the lab samples. For data, see Table S2

The decision on which categories to compare was based on the PCA results (Figure 1). Categories were’Fieldsamples’ and ‘Labsamples’ independent from temperature and origin for ‘rearing condition’, ‘Spitsbergen’ and’Helgoland’ within the lab and the field samples independent from temperature for ‘origin’ and ‘5’, ‘10’ and ‘15’ for ‘Temperature’ within lab samples by origin. To estimate the number of sites that differ in methylation state between lab and field samples, between origins in the lab and field samples and between origins in the lab samples per temperature, we applied differential methylation analyses using the R package ‘DESeq2’ V 1.24.0 (Love et al., 2014), ‘IHW’ to correct p‐values according to independent hypothesis filtering (Ignatiadis et al., 2016) and ‘VennDiagram’ (Chen & Boutros, 2011). Outliers were kept, and IHW was run with *p*‐adj < 0.05 (Benjamini & Hochberg, 1995; Kassambara, 2020).

### 2.10 GO term analysis

We tested for enrichment of gene ontology terms (biological process (BP), cellular component (CC) and molecular function (MF)) in genes that were differentially expressed between origins or temperatures with the R package ‘topgo’ (Alexa & Rahnenführer, 2010). GO terms were transferred from the annotations obtained from the ORCAE database (Sterck et al., 2012), accessed on May 29 2020. Significance levels for GO term enrichment were adjusted with the Benjamini and Hochberg correction (Benjamini & Hochberg, 1995)⁠ to control for the false discovery rate when conducting multiple comparisons. We reduced redundancy in the enriched GO terms with REVIGO (Supek et al., 2011), using a threshold of 0.7 (semantic similarity SimRel, using the whole UniProt database). We further simplified the GO terms with the function simplifyGOterms of the compEpiTools R package (v3.13, and Kishore et al., 2015), considering the parent GO term as redundant if the two gene sets overlapped by more than 30% of the parent genes.

## 3 RESULTS

Of the 476.16 million high‐quality reads of all samples (Table S1, TrimmedReads, Column C), 10.94 million mapped back against the *S*. *japonica* reference genome (Column D), but only 4.02 million of these mapped uniquely (Table S1, Column E). For all sequenced reads that we obtained, this gives a mapping level of 1.00 ± 1.02% (mean ± SD, uniquely mapped reads divided by trimmed reads times 100; Table S1, Column F), but for *Saccharina latissima* sequences (only taking those reads into account that actually mapped back to the reference genome) it gives a unique mapping level of 41.31 ± 4.76% (mean ± SD, uniquely mapped reads divided by mapped reads times 100, Table S1, Column G). However, only those reads that mapped uniquely and had a coverage ≥3 were taken into further analyses. This gives an overall cytosine methylation coverage level (number of methylated sites covered by ≥3 reads, divided by the number of all potential methylation sites (in silico digested MethylRAD tags) times 100) of 0.21% ±0.13% SD, with 0.03% minimum and 0.75% maximum coverage (see Table S4).

### 3.1 PCA

Samples were most distinct in their methylation patterns between rearing conditions (lab vs. field, Figure 1a), regardless of the origin of the samples, followed by differences between the origins in the field samples (Figure 1a). Along the first two principal components, which explained 79.3% of the variation in methylation level, samples formed three major groups: (1) Spitsbergen field, (2) Helgoland field and (3) all lab samples (Figure 1a). Within the lab samples, we further classified/grouped ‘temperature’ and ‘origin’ levels (Figure 1b,c). The three ‘temperature’ groups (5°, 10° and 15°) were not distinct, but overlapped (Figure 1b). For ‘origin’, lab samples divided into two distinct groups, Spitsbergen and Helgoland, which could explain 82.7% of the variation in methylation level (Figure 1c).

### 3.2 DNA methylation levels

**FIGURE 2:**
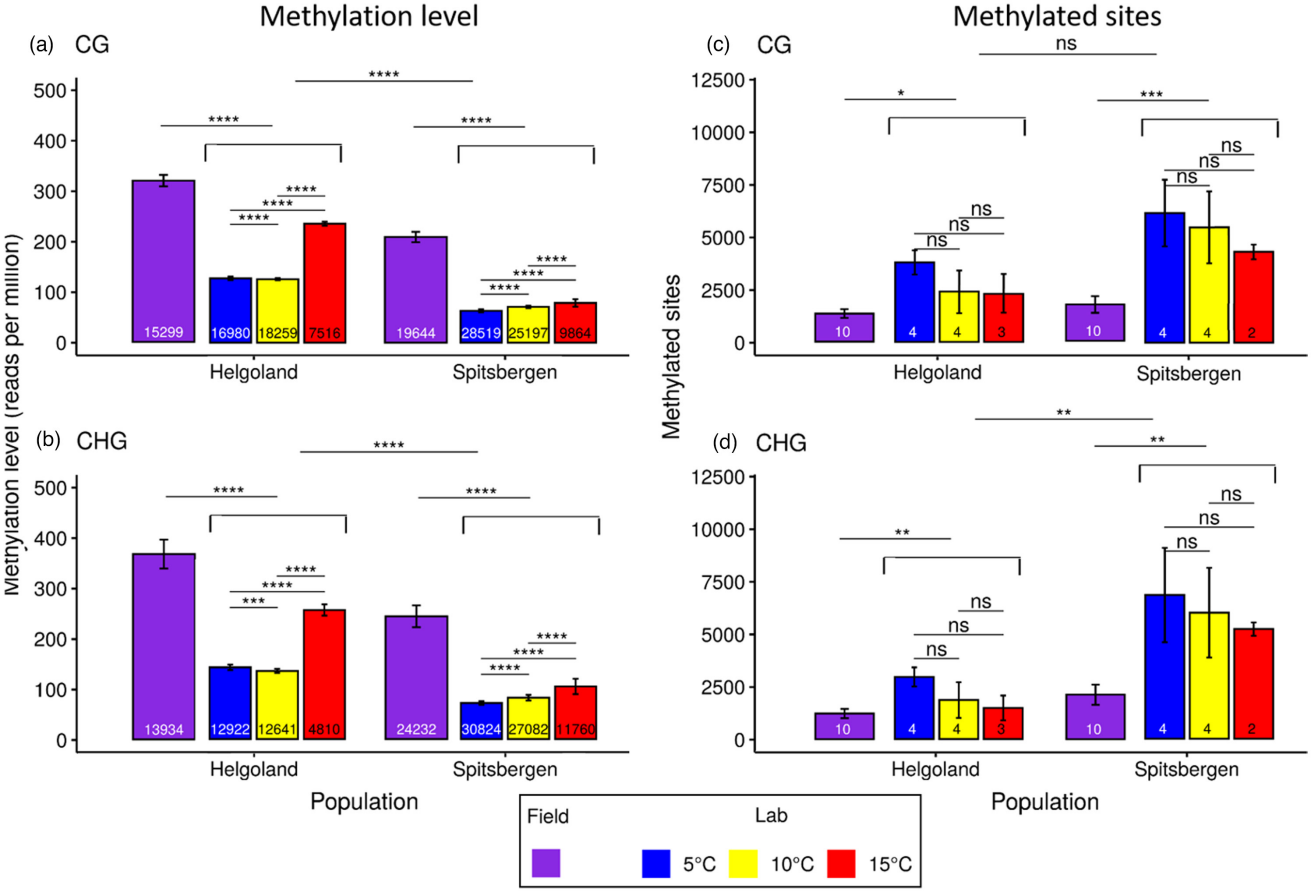
DNA methylation level (a, b) and number of methylated sites (c, d) in the genome of *Saccharina latissima* sporophyte lab samples from Helgoland and Spitsbergen; shown in CG (a, c) and CHG (b, d) sequence contexts under 5°C (blue bars), 10°C (yellow bars) and 15°C (red bars) rearing temperature, and in field samples (violet bars). Significance codes after *p*‐value correction (Benjamini–Hochberg) for multiple comparisons: ‘<0.05’: *, ‘<0.01’: **, ‘<0.001’: *** and ‘<0.0001’: ****. The numbers at the bottom of the bars refer to the number of methylated sites for which the methylation level was estimated (a, b), and to the number of sporophytes for which the number of methylated sites was estimated (c, d) respectively

**FIGURE 3:**
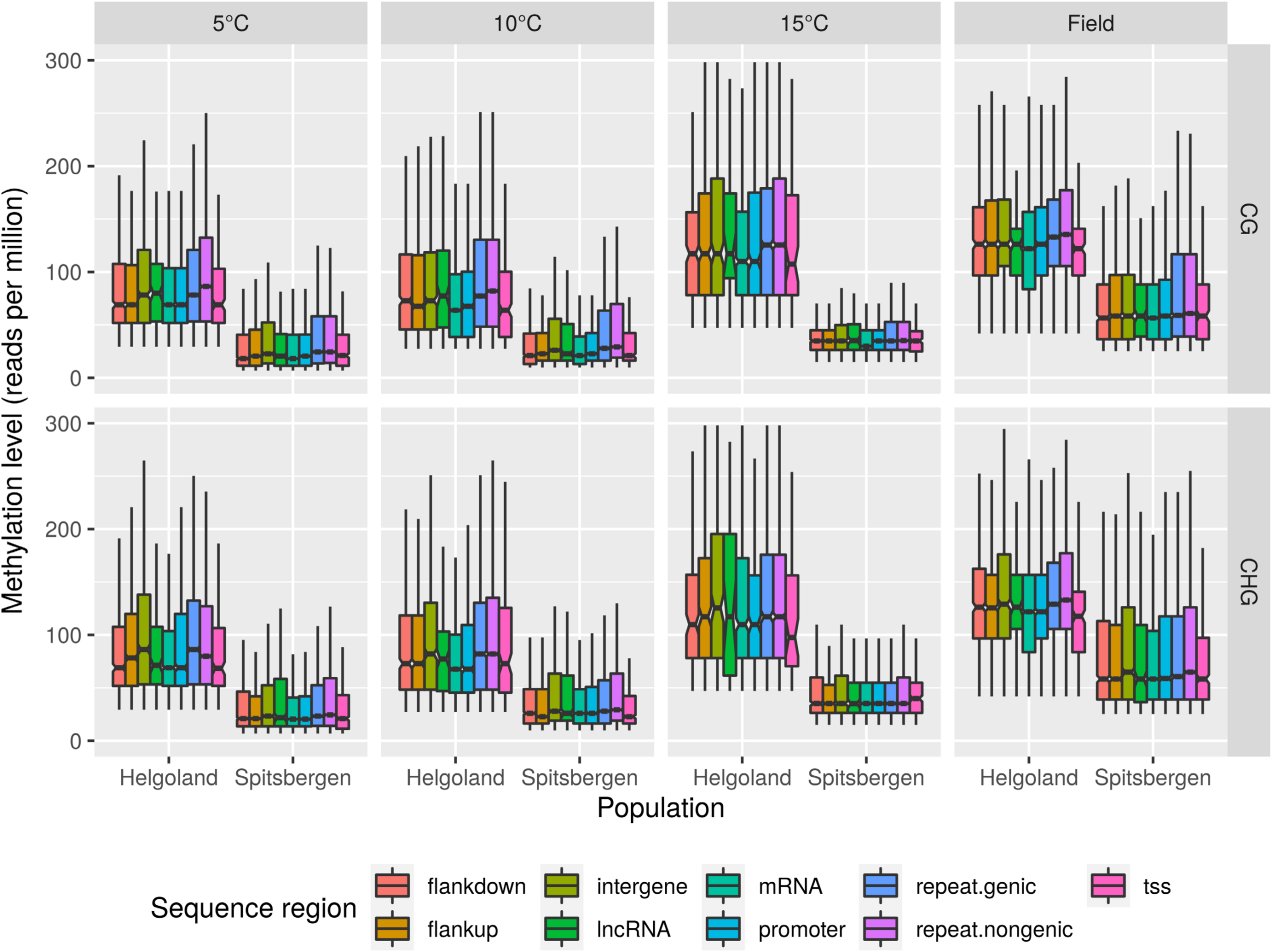
DNA methylation level in CG and CHG sequence contexts throughout the genome of *Saccharina latissima*, shown for lab cultures raised under different temperatures (first 3 columns) and in sporophyte field samples (last column) from Helgoland and Spitsbergen. The boxes show the interquartile ranges (IQR), and the whiskers extend 1.5 times the IQR from the box margins. Outliers are not shown. Notches represent the 95% confidence interval around the medians. If two boxes’ notches do not overlap, their medians differ with 95% confidence; flankup: 10 kbp upstream of genes (protein coding genes or lncRNAs); flankdown: 10 kbp downstream of genes (protein coding genes or lncRNAs); intergene: intergenic region; lncRNA: long noncoding RNA gene; mRNA: protein coding gene; promoter: 2 kbp upstream to 200 bp downstream of the annotated transcription start site; repeat.genic: annotated transposable element in a gene region; repeat.nongenic: annotated transposable element in an intergenic region; tss: transcription start site extending 300 bp upstream to 50 bp downstream of the annotated transcription start site. See Table S5

Methylation levels (reads per million) in both contexts (CG and CHG) were more than twofold higher in field samples than in lab samples (*p*‐value < 0.0001 (Benjamini–Hochberg) Figures 2a,b and 3). Furthermore, Helgoland samples showed significantly higher methylation levels than Spitsbergen samples for both rearing conditions (field and lab, *p*‐value < 0.0001 (Benjamini–Hochberg), Figure 2a,b). Between Helgoland and Spitsbergen, there were slight differences regarding the correlation of methylation level to temperature in the lab samples: In the Spitsbergen lab samples, methylation levels increased significantly with temperature (*p*‐value < 0.0001 (Benjamini–Hochberg) Figure 2a,b). However, in the Helgoland lab samples, methylation levels were lowest at 10°C and increased significantly under 15°C, as well as under 5°C (*p*‐value < 0.0001 (Benjamini–Hochberg), Figure 2a,b). <0.00001 to <0.028, Figure 3).

At any temperature, DNA methylation levels were significantly higher in Helgoland samples than Spitsbergen across the entire genome (*p*‐value < 0.0001 (Benjamini–Hochberg), Figure 2a,b), and in all examined sequence contexts (Figure 3). Within a ‘cohort’ (same origin and temperature, e.g. ‘5°C Helgoland’, Figure 3), methylation levels differed significantly between sequence regions. In Helgoland samples, methylation levels were highest in repetitive DNA sequences (transposable elements) inside and outside of genes (repeat.genic and repeat.nongenic, *p*.adj. << 0.00001 to <0.028, Figure 3, *p*‐values see Table S5) and intergenic regions (intergenic, *p*.adj. << 0.00001 to <0.0006), and lowest in mRNA‐coding regions (mRNA, *p*.adj. << 0.00001 to <0.001; Figure 3). In Spitsbergen samples, methylation levels were more evenly distributed across all sequence regions, particularly at 15°C. At 5 and 10°C, transcription start sites (tss) and mRNAs (mRNA) showed significantly lower methylation than any other sequence region (*p*.adj < 0.00001 to <0.027, both, Figure 3). In contrast, repetitive DNA sequences outside of genes (repeat.nongenic, *p*.adj. << 0.00001 to <0.017) and intergenic regions (intergene, *p*.adj << 0.00001 to <0.049) showed significantly highest methylation levels of all sequence regions.

### 3.3 Number of methylated sites

To distinguish between the genome‐wide level of methylation, and the spread of this methylation across the genome, we analysed not only methylation levels but also methylated sites. In general, where we recorded increased methylation levels, we often counted a lower number of methylated sites, which means that when methylation increased, this focused on a core region of the genome and could not be explained by an increased number of methylated sites.

**FIGURE 4:**
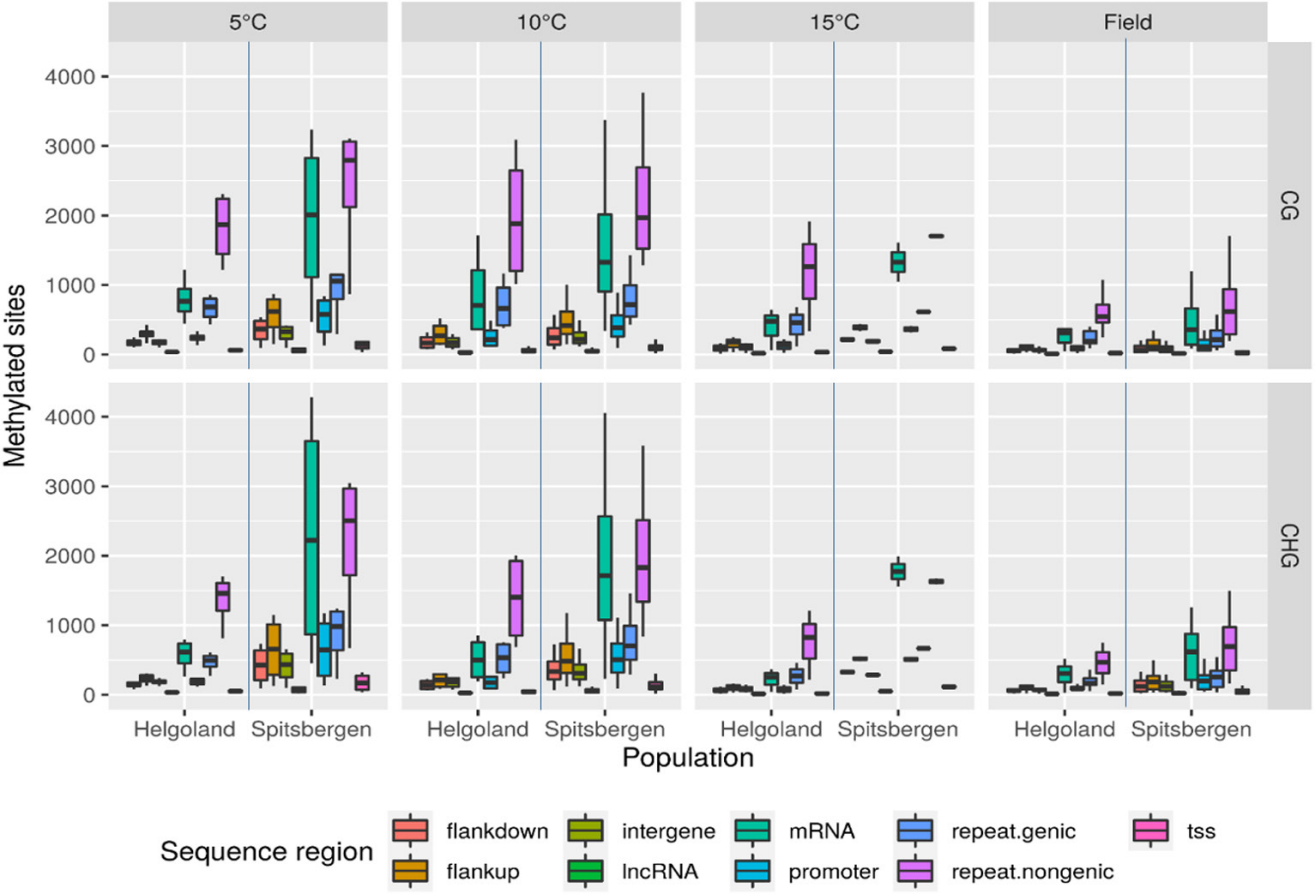
Number of methylated sites in CG and CHG sequence contexts throughout the genome of *Saccharina latissima*, shown for lab cultures raised under different temperatures (first 3 columns), and in sporophyte field samples (last column) from Helgoland and Spitsbergen. Flankup: 10 kbp upstream of genes (protein coding genes or lncRNAs); flankdown: 10 kbp downstream of genes (protein coding genes or lncRNAs); intergene: intergenic region; lncRNA: long noncoding RNA gene; mRNA: protein coding gene; promoter: 2 kbp upstream to 200 bp downstream of the annotated transcription start site; repeat.genic: annotated transposable element in a gene region; repeat.nongenic: annotated transposable element in an intergenic region; tss: transcription start site extending 300 bp upstream to 50 bp downstream of the annotated transcription start site. See Table S6

The number of methylated sites was significantly higher in lab samples from Spitsbergen than from Helgoland (*p* < 0.05, *p* < 0.01, Figure 2c,d). The number of methylated sites did not differ significantly between temperatures, but showed a downward trend (Figure 2c,d). The number of methylated CG sites showed no significant difference between origins (Figure 2c), but between lab and field samples for both origins (*p* < 0.05 and <0.001, (Figure 2c)). However, at CHG sites the number of methylated sites differed significantly between origins (*p* < 0.01, Figure 2d), and between lab and field samples (*p* < 0.01, Figure 2d). Regarding sequence regions, both origins showed the lowest number of methylated sites in intergenic regions (Figure 4, ‘intergene’, nearly no sites), followed by regions 10kbp up‐ and downstream of genes (‘flankup’ and ‘flankdown’) and transcription start sites (‘tss’, Figure 4). In contrast, non‐coding repetitive DNA (‘repeat.nongenic’) and mRNA coding regions (‘mRNA’) showed the highest numbers of methylated sites in both origins and rearing conditions (Helgoland and Spitsbergen, field and lab, Figure 4).

At 5°C, Helgoland lab samples showed significant differences in the number of methylated sites between most of the examined sequence contexts (28 significant tests of 36 pairwise comparisons, range of *p*.adj <0.01 to <0.036, see Table S6). At 10°C, only a few (8 of 36) sequence contexts showed significantly different numbers of methylated sites, mostly comparisons with repeat.nongenic contexts (five of eight, *p*.adj. <0.035 to <0.05). At 15°C, the number of methylated sites did not differ among any sequence contexts. The Helgoland field samples showed highly significant differences in the number of methylated sites among sequence contexts (27 significant of 36 pairwise comparisons). Most methylated sites were located in non‐genic repeat regions (*p*.adj. << 0.00001 to any other sequence contexts) and mRNA (*p*.adj. <0.002 to <0.02); least sites were methylated in transcription start sites (TSS, *p*.adj. <0.0001 to 0.002).

In contrast to the Helgoland samples, the Spitsbergen samples showed most significant differences in the number of methylation sites between sequence contexts at 15°C (7 of 11 comparisons with repeat.nongenic, all *p*.adj << 0.0001). At 10°C, only two differences were observed (both in comparison with repeat.nongenic, *p*.adj. <0.031 and <0.036), and at 5°C eight significant differences (six of which in comparison with repeat.nongenic, *p*.adj. <0.015 to <0.047).

### 3.4 Sequence context of differentially methylated sites

**FIGURE 5:**
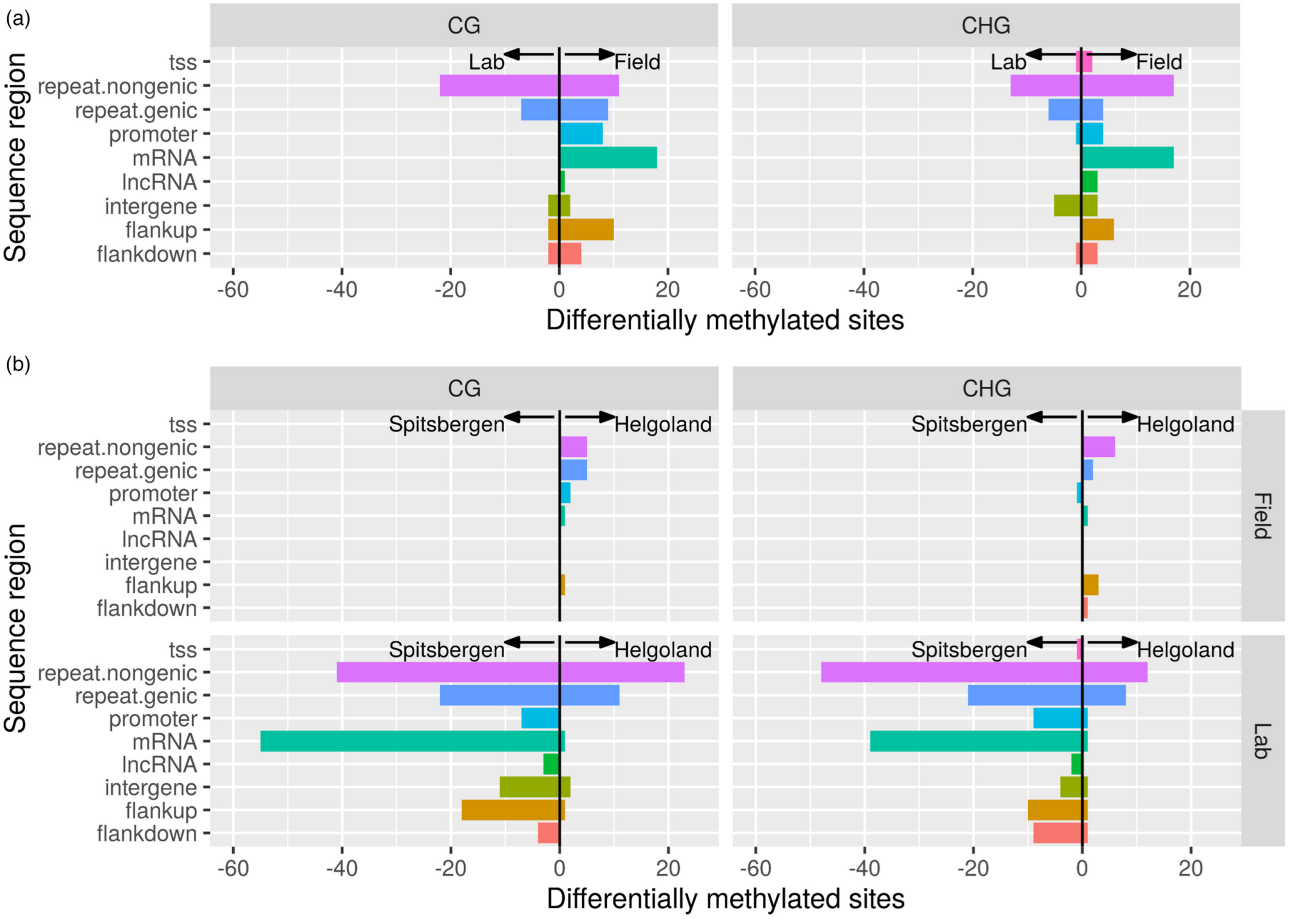
Number of methylated sites in CG and CHG context of nine different sequence regions that were differentially methylated (*p*.*adj* < 0.05) between (a) sporophyte field samples and lab cultures of *Saccharina latissima*, and (b) between samples from Helgoland and Spitsbergen in sporophyte field samples (upper row) and lab cultures (lower row). The sites with higher methylation levels in the field (a) or Helgoland (b) samples appear as positive counts, the sites with higher methylation levels in lab (a) or Spitsbergen (b) samples as negative counts; flankup: 10 kbp upstream of genes (protein coding genes or lncRNAs); flankdown: 10 kbp downstream of genes (protein coding genes or lncRNAs); intergene: intergenic region; lncRNA: long noncoding RNA gene; mRNA: protein coding gene; promoter: 2 kbp upstream to 200 bp downstream of the annotated transcription start site; repeat.genic: annotated transposable element in a gene region; repeat.nongenic: annotated transposable element in an intergenic region; tss: transcription start site extending 300 bp upstream to 50 bp downstream of the annotated transcription start site. See Tables S7 and S8

Between lab and field samples, most of the differentially methylated sites were observed for nongenic repetitive contexts (repeat.nongenic) and for coding regions (mRNA, higher methylation levels in field samples, Figure 5a). Lab samples showed a higher number of differentially methylated sites between origins than field samples, particularly in CG sites (Figure 5b). These differences were dominated by increased methylation in Spitsbergen samples, mostly in repeat regions and coding regions (mRNA).

### 3.5 GO terms

In the field samples, only samples from Helgoland showed enrichment of functions in hypermethylated sites (Figure S4: a1–3). Hypermethylated sites in gene bodies (mRNA) were enriched for functions related to gene expression (GO:0045892, GO:003714 and GO:003712; Figure S4: a1). Noncoding regions with hypermethylated sites were flanking genes involved in photosynthesis (GO:0015979 and GO:19684; Figure S4: a1), mRNA (GO:0006397 and GO:008380; Figure S4: a1), electron transport chain (GO:0022900; Figure S4: a1), chloroplast, thylakoid membrane (GO:0044434, GO:0009535, GO0044436 and GO:0009579 Figure S4: a3), plastids (0044435; Figure S4: a3) and oxidoreductase activity (GO:0016730; Figure S4: a3).

Spitsbergen lab samples showed GO terms enriched in bodies of genes (mRNA regions) involved in nucleoside metabolism (GO:0009186, GO:0009138, GO:000933 and GO:0006233; Figure S4: b1), phytochrome synthesis (GO:0051202 and GO:0010024; BP domain), cell structure and organelle movement (GO:0015629, GO:0000808 and GO:0016459; Figure S4: b2), NELF complex (GO: 0032021; Figure S4: b2) and in DNA assembly (GO:0050897, GO:0016636, GO:0004329, GO:0017076, GO:0032559 and GO:1901265; Figure S4: b3). In flanking regions, Spitsbergen lab samples showed hypermethylation in genes involved inter‐ and intracellular transport (GO:0006612, GO:0071806, GO:0065002 and GO:0035494; Figure S4: b1), chorismate metabolic process (GO:0046417; Figure S4: b1) and DNA repair (GO: 0036297; Figure S4: b1).

Helgoland lab samples showed hypermethylation only in flanking regions, of genes involved in metabolic processes (organelle and ATP synthesis, cell membrane components (GO:0006779, GO:0006289, GO:0046854, GO:0022900 and GO:0033013; Figure S4: b1)), chloroplast components (GO:0044435, GO:0044434, GO:0009535, GO:0044436 and GO:0009579; Figure S4: b2) as well as DNA/RNA component and pigment assembly (GO:0008441, GO:0016730, GO:0008252, GO:0004109 and GO:0016634, Figure S4: b3).

Only one term was significantly enriched in hypermethylated sites of lab samples when compared with field samples (nonmotile cilium assembly, GO:1905515; BP; Figure S5). All other terms were significantly enriched in hypermethylated sites of the field samples, predominantly in the bodies of genes involved in organelle functioning (GO:0007033 and GO:0007040), macromolecule/protein modification (GO:0006470, GO:0006464, GO:0071586 and GO:0043412), amino acid synthesis (GO:0009082) and transcription (GO:0045131, GO:0004160, GO:0016791, GO:0036002, GO:0003918, GO:0042578, GO:0003993, GO:0005086 and GO:0061505; Figure S5).

Terms enriched in hypermethylated sites of flanking regions, again only in the field samples, were related to mRNA methylation (GO:0080009), modification (GO:0016556), splicing (GO:0000381 and GO:0000380) and regulation of acetate metabolism (GO:0019427, GO:0006083 and GO:0019222) or other metabolisms (GO:0050789 and GO:0060255; Figure S5).

### 3.6 Microbial contamination

Based on the 14.12 million high‐quality reads from the representative sample, only 45% mapped against the *S*. *japonica* genome, suggesting that ca. 55% of the extracted DNA must originate from other than the target species. Indeed, 46.3% of the high‐quality reads were taxonomically annotated as *Proteobacteria*, another 1.4% as undefined eukaryotes (Figure S6). Thus, of the 55% of the high‐quality reads that did not map back to the *S*. *japonica* genome, ca. 48% represent predominantly bacterial contamination, leaving 55 − 48% = 7% of the reads that did not originate from *S*. *japonica* as undefined.

### 3.7 Methylome differences between origins

We found the methylome of *Saccharina latissima* to be origin specific, with significant differences between Helgoland and Spitsbergen samples (field and lab) in genome‐wide methylation level (Figures 2 and 3), and in number of methylated sites (Figures 2 and 4, Figure S7), and with significant differences in methylation at specific sites (Figure 5, Figure S8). When comparing the field samples by origin, very low numbers of differentially methylated sites were observed; the methylation level was similar at most sites when comparing Helgoland and Spitsbergen. Those sites showing significant differences in methylation levels between origins were nearly exclusively found to be higher methylated in the Helgoland samples (Figure 5b), and occurred mainly in repeat regions. In lab samples, most of the sites that were significant differentially methylated between Spitsbergen and Helgoland samples occurred in the sequence contexts ‘mRNA’ and ‘repeat.nongenic's (Figure 5b), corresponding to the sequence regions showing the highest number of methylated sites (Figure 4). No differentially methylated sites were detected for ‘lncRNA’, ‘tss’, ‘intergene’ (both CG and CHG) or ‘flankdown’ (in CG context). Methylation was highly site‐specific across origins, as despite these regions having methylation levels similar to the other regions examined (Figure 3), the number of methylated sites was very low (Figure 4).

### 3.8 Methylome variation by temperature

Compared with ‘rearing condition’ (= lab vs. field) and ‘origin’ (Spitsbergen vs. Helgoland), ‘rearing temperature’ was the factor least able to explain methylation patterns of our samples. The methylomes of both origins responded similarly, with an increase in genome methylation in response to rising temperatures. Origins only showed four sites of significantly different methylation level at the corresponding temperatures (see Figure S8). For both sequence contexts (CG, CHG), Helgoland samples showed lowest methylation levels at 10°C and highest levels at 15° (5°C > 10°C < 15°C), while methylation level of the Spitsbergen samples was positively correlated with temperature (5°C < 10°C < 15°C, Figure 2a,b). Even though a trend of negative correlation in the amount of methylated sites to temperature was observed for both origins and both sequence contexts, the differences between temperatures were not significant because of high variation among the few samples (Figure 2). The sequence regions ‘lncRNA’ and ‘tss’, followed by ‘flankup’, ‘flankdown’, ‘intergene’ and ‘promoter’ appear to be the regions that are most responsive to changes in ambient temperature: While they showed methylation levels similar to the other examined sequence regions (Figure 3), methylation was restricted to only few (<500 to <250) core sites (Figure 4).

## 4 DISCUSSION

Our study confirms that sugar kelp indeed has a methylome, with important ecological implications. While we hypothesized that sample origin would explain most of the methylome variation in our samples, our findings showed that cultivation had a stronger effect on methylome patterns than expected. This suggests that farmed kelp originating from hatcheries, or grown in the lab, differs fundamentally in epigenetically controlled characteristics from wild kelp of the same origin. Epigenetic mechanisms have the potential to trigger genomic changes via transposition through activation of transposable elements (Ramakrishnan et al., 2021), and thus, can provide an accelerated pathway for evolutionary change and adaptation.

### 4.1 Peculiarities of the sugar kelp methylome

In *Saccharina latissima*, our results suggest a cytosine methylation coverage level of 0.21 ± 0.13% (mean ± SD, see Table S4), ranging from 0.02% to 0.75%. This is lower than reported for the congener species *S*. *japonica* (1.4% methylation level for CG, CHG and CHH context, sporophytes and gametophytes, based on whole‐genome bisulfite sequencing (WGBS), Fan et al., 2020). However, the methylation level observed in our study is expected to be lower than reported for *S*. *japonica* owing to the fact that we targeted the specific bases within the degenerate adaptor endings 5′‐NNNT‐3′ and 5′‐NNNC‐3′, which only constitute about one‐eighth of all CG and CHG sites (Wang et al., 2015). Thus, the overall methylation levels of *S*. *latissima* can be expected to be on average eight times higher than the mean number of covered sites we had obtained, namely 1.66%, ranging from 0.18% to 6% (Table S4, Column O) and, thus, are comparable to *S*. *japonica*. Furthermore, the MethylRAD protocol (Wang et al., 2015) cannot identify methylation in CHH sequence contexts, which were reported to be the dominant type for cytosine methylation in *S*. *Japonica* (Fan et al., 2020). Hence, it is likely that the total methylation level in *S*. *latissima* will be similar to that of *S*. *japonica* when analysed with a protocol that targets all methylation sites, such as WGBS.

### 4.2 Cultivation aspects

Cultivation had the strongest influence on the methylome of *Saccharina latissima*, as field and lab samples clustered separately (Figure 1a) regardless of origin and temperature. Even though the similarities within lab and field samples might have been a batch effect (lab and field samples were sequenced on separate flow cells for logistical reasons), we presume this would have affected the batches randomly. Still, given this is logistically possible, lab and field samples should be sequenced in mixed batches to prevent any batch effects. However, we observed higher methylation levels (Figures 2 and 5a), but simultaneously less methylated sites (Figure 2) in field samples compared with lab samples in the same respective sequence contexts, which we argue dismisses possible batch effects. The observed pattern suggests that while few core genes are strongly silenced, more genes are expressed in the wild origin, as methylation level and gene expression were shown to be inversely correlated in kelp (Fan et al., 2020). In contrast, the lab samples experienced stable abiotic factors (temperature, airflow, water velocity and nutrient supply), with only the light oscillating in 18/6h light/dark cycles but with constant irradiance during light. The similarity in the methylome among lab samples, and their contrast to field samples (Figure 1), independent of temperature or origin, in addition to the nearly complete lack of hypermethylated genes (Figure S5) suggests that lab conditions impose a certain epigenotype that can be expected to affect the performance of lab‐grown cultures. Differences between lab and field sporophytes that have been observed in transcriptomic analyses further support this hypothesis (Heinrich et al., 2016). For lab experiments that focus on responses from the sampled wild populations, our results suggest that spores should be obtained only shortly before the start of a respective experiment when growing sporophytes from gametophyte cultures. Furthermore, for kelp farming, the origin seems to be the critical factor that needs to be matched with hatchery conditions.

In agri‐/mariculture, priming – the exposure to a stressor eliciting epigenetic (stress‐) responses (Holeski et al., 2012; Jueterbock et al., 2021) – is used to enhance tolerance of or resilience to responses to this stressor. Specimens from both origins responded to rising temperatures with an increase in DNA methylation but a decrease in the number of methylated sites (Figures 2, 3, 4). Given the negative correlation between DNA methylation and gene expression (Fan et al., 2020), this suggests that certain genes are effectively silenced, while others are released from their inactive state. Even though heat stress was never encountered during our experimental setup, sea water temperatures in Helgoland can reach the upper thermal tolerance limit for *S*. *latissima* (Bolton & Lüning, 1982, Lüning, 1984; see Figure S2). Low levels of methylation spread across the genome appear to reflect a low stress environment (5°C for Spitsbergen and 10° for Helgoland). The increase in cytosine methylation levels of certain regions and genes seems to be a direct response to a deviation from epigenetically inherited memories of ambient temperature. This is reflected by an increase in DNA methylation with increasing temperature in the samples from Arctic Spitsbergen, and a significant increase in DNA methylation in the samples from temperate Helgoland in response to both higher (15°C) and cooler (5°C) than the long‐term ambient water temperatures; even though the response to 15°C was much stronger (Figure 2). The methylation sites responding to increasing temperatures did not correspond between the two origins.

### 4.3 Silencing of transposable elements

A huge proportion of the genome (40% in *S*. *japonica*; Ye et al., 2015) is made up of repetitive noncoding DNA regions (transposons). Methylation levels in this type of sequence region (repeat.nongenic) were comparable with other examined regions (Figure 3), while the number of methylated sites varied between samples (Figure 4), and featured a high number of differentially methylated sites when comparing lab and field samples (Figure 5a), or lab samples from Helgoland and Spitsbergen (Figure 5b). In contrast to the general pattern observed (increase in methylation level, but decrease in number of sites), regions coding for transposable elements were high in level and number of sites. Cytosine methylation is well known to be essential for silencing transposable elements (Takuno et al., 2016; Zhang et al., 2006), and to regulate gene expression (Bender, 2004). Furthermore, silencing of promoter regions and gene body via DNA methylation has been shown to be an additional tool for silencing transposons in plant genomes (Zhang et al., 2006). CHH here is the dominant methylation context in transposons in plants, while CG predominates in gene bodies (Bender, 2004; Boquete et al., 2021; Schmitz et al., 2019). In our study, *S*. *latissima* origins showed a positive correlation of CG methylation level with temperature. In addition, methylated sites decreased with increasing temperature, hence became more site specific. This highlights both regions presumably important for heat stress priming, and the putative role of DNA methylation in controlling gene expression during acclimation or even adaptation processes. As the MethylRAD protocol does not assess CHH methylation, which is the sequence context found to be dominantly methylated in transposons in plants (Dubin et al., 2015), the absolute genome‐wide transposon methylation can be presumed to be higher. As transposable elements, both within and outside of genes, constituted the sequence regions that were different in methylation level, this again highlights the importance of cytosine methylation in controlling these genomic regions (Gehring & Henikoff, 2007), with respective levels likely higher if CHH regions had been included in our protocol (see section ‘peculiarities of the sugar kelp methylome’). Furthermore, CHH methylation has been observed to be highly influenced by temperature (Dubin et al., 2015), hence the patterns we observed without analysing CHH likely are only a rough assessment of the temperature effect.

### 4.4 Microbial contamination/biome

A large portion of methylations obtained from the extracted DNA stemmed from bacteria (~46%, Figure S6b). We believe this may be partly due to a higher resistance of algal cells to lysis during DNA extraction, resulting in a preferential DNA extraction from microbiome/contaminant cells, even when those only made up a minor component in terms of biological material. Of the extracted and sequenced total DNA (high‐quality reads), 46.3% were taxonomically annotated as proteobacteria and another 1.4% as undefined eukaryotes (Figure S6). In the sequenced MethylRAD tags, up to 99% of the originally sequenced reads were presumably stemming from other than the target species, suggesting that the MethylRAD protocol favoured contaminated DNA over DNA from the target species, presumably in the amplification steps of the protocol. However, we exclusively analysed those sequences that had uniquely mapped to the target genome. Thus, despite at a high level, microbial contamination does not affect the identified methylation patterns of our analyses.

### 4.5 Ecological implications

Abiotic and biotic stressors are known to alter epigenetic marks (Erdmann & Picard, 2020). A possible explanation for the significant differences in methylation level between origins in all sampled conditions might be that the Helgoland origin responded more strongly to elevated temperature than the Spitsbergen origin due to the build‐up of a transgenerational priming memory as it encountered heat stress in previous generations (see Figure S2). In contrast, the Spitsbergen origin very rarely encountered temperatures above 7°C (see Figure S3) and, thus, lacks a priming memory related to heat stress that may allow for a faster or stronger response (Jueterbock et al., 2021). This might explain the differences observed for the methylomes’ reactions towards temperature, and the differences between the wild origins. While sea surface temperatures seldom exceed 7°C in the high Arctic during summer, they can get close to the species’ lethal temperature limit of ~22°C (Bolton & Lüning, 1982) during exceptionally hot summers in Helgoland. The frequency of these exceptional summers increases due to climate change. Hence, heritable epigenetic markers such as cytosine methylation reacting to heat stress with an increase in methylation (Wibowo et al., 2016) may accumulate in the temperate origin via transgenerational priming, while remaining absent in the Arctic origin. Given genome‐wide methylation levels correspond to stress intensity, the low genome‐wide methylation levels of the Spitsbergen origin likely indicate that cold temperatures of up to ~5°C represent a low‐stress environment for *S*. *latissima*. Sporophytes from Helgoland that only seasonally experience temperatures of ~5°C seem to be more sensitive to changing temperatures (both towards colder and warmer), and hence react with changes in epigenetic regulation patterns towards both colder and warmer than the long‐term ambient temperature (‘Helgoland’, Figures 2, 3, 4, 5).

The abiotic environment of the high Arctic has often been considered to be a challenge for perennial primary producers such as kelp (Becker et al., 2009; Karsten et al., 2001; Raven et al., 2002; Zacher et al., 2009, 2016). However, with the expansion of kelp forests towards the north, and their retreat from the south, it becomes clear that boreal‐temperate kelp such as *S*. *latissima* possess a high plasticity especially in cold environments, and at least the Arctic origin shows a high affinity towards its cold environment. Lowest methylation levels in the Spitsbergen origin at 5°C possibly reflect eco‐phenotypic adaptation to low temperatures. In *Arabidopsis*, DNA methylation correlated positively with latitude in CG contexts, and negatively with temperature in transposable elements, which was presumed to indicate the preference of this species towards warmer temperature conditions (Kawakatsu et al., 2016). Our findings show the inverse pattern in the methylome of *S*. *latissima*. The methylation level appears to decrease with increasing latitude, and accordingly increases with increasing temperature, especially in transposable elements. The correlation between DNA methylation (in different contexts and sequence regions) and temperature appears to be an indicator for the species’ optimal temperature regime. Hence, the observed climate change‐induced shifts towards the north of this amphi‐boreal species (Guzinski et al., 2020; Jueterbock et al., 2013), plus the correlation between methylation patterns and temperature we obtained for *S*. *latissima*, especially in the Arctic origin, are a good indicator for the high affinity towards cold adaptation in this species.

### 4.6 Eco‐phenotype

Methylomes in our study showed origin‐specific signatures (Figures 1, 2, 3, 4, 5, Figure S4). However, attempts at distinguishing origins in *S*. *latissima* only recently gained momentum, and until now are centred on the genotype approach. Genetic differences among origins of *S*. *latissima* so far have focused on cytochrome oxidase I (COI), SNPs and microsatellites (Guzinski et al., 2020; Møller Nielsen et al., 2016; Neiva et al., 2018; Paulino et al., 2016). However, in plants, COI has been recognized as unsuited for detecting differences below species level due to the low substitution rate of mitochondrial DNA (Hollingsworth et al., 2009). Instead, specific mini‐barcodes (amplicons of 120–220 bp length, Little, 2014) seem to be better suited for the DNA barcoding purpose in brown macroalgae (Ortega et al., 2020), and microsatellites specifically tested for the respective macroalgal species will help to assess within‐species variation (Guzinski et al., 2016; Neiva et al., 2018; Paulino et al., 2016). In *Saccharina latissima*, the resolution of genetic variation along the European coast ranges from differences between northern and southern European origins (rough border Denmark, COI; Neiva et al., 2018), over (in silico) origin distinction within larger regions based on genotype (microsatellites, Neiva et al., 2018; Paulino et al., 2016), to differences in within‐origin genetic diversity based on SNPs (Guzinski et al., 2020). In contrast to these genotype approaches to origin distinction, our results suggest that assessment of common‐garden methylome differences appear to provide another layer of molecular distinction between origins. According to our results, origin and source (growing from cultured vs. wild gametophytes) seems to play an important role. Short‐term within‐generation acclimations, e.g. known as ‘epigenetic induction’ (Jablonka & Raz, 2009) or ‘priming’ (Holeski et al., 2012), might become heritable traits (epigenetic inheritance). Previous observations on increased physiological performance and heat tolerance after heat exposure in *S*. *latissima* further support this theory (Harley et al., 2012). However, additional research is necessary to assess to what degree the population differences in methylome patterns are driven by or independent from underlying genetic differences.

Regarding local adaptation, earlier studies on intraspecific variation in *S*. *latissima* offer both phenotypic plasticity (Schlichting, 1986) and local adaptation of genetically distinct ecotypes (Gerard, 1988; Gerard & Du Bois, 1988), as explanation for the broad differences observed, e.g. in the habitus of this species. As neither ecotype nor phenotype fully acknowledge the important role of heritable epigenetic mechanisms for local adaptation, we feel a more adapt term would be that of ‘eco‐phenotype’: the combination and interaction of both genetic (DNA sequence related) and epigenetic mechanisms that lead up to a specific ‘adaptation’ of single individuals or origins to their respective environment, which can be passed on to following generations, and typically prevails for at least one generation when this generation encounters factors differing from those of the parental generation. With this, the definition of ‘adaptation’ would deviate from the classical one (‘heritable, but DNA sequence‐based’) towards ‘heritable, but transcription‐based’, as it would include epigenetic mechanisms influencing gene expression.

## 5 CONCLUSION

The significant difference in the methylomes of *Saccharina latissima* from field samples of two origins supports the hypothesis that DNA methylation is involved in ecotypic differentiation/local acclimation. Furthermore, it supports the hypothesis that DNA methylation, due to its potentially heritable character, seems to be crucial also for long‐term adaptation to local factors, similar to evolutionary processes, but without alteration of the gene sequence. Hence, we presume it is valid to consider DNA methylation in kelp as important driver underlying eco‐typic differentiation.

Between Arctic and temperate origins, samples from the field sites, and to a larger extent lab samples, showed clearly distinguishable, differentially methylated sites throughout the methylome. This molecular variation, and its resulting variation in phenotype and physiology should be taken into account for modelling studies (e.g. physiological responses to biotic and abiotic factors, distribution), which until now typically considers the whole species as a homogeneous ‘unit’. Differences in methylation patterns as well as physiological responses between origins indicate that there is no ‘unit’ (Diehl et al., 2021; Heinrich et al., 2016; Monteiro et al., 2019). Instead, eco‐physiological parameters, for example, should be specifically matched to the region/origin of *Saccharina latissima* they were obtained for, instead of putting them as a ‘global response’ of the species. Furthermore, populations living in waters that remain cool throughout the year without much thermal fluctuation should be regarded distinct from those living in warmer temperatures with higher seasonal fluctuations. As this publication is the first to tentatively compare the methylomes of different origins within a macroalgal species, further research will be crucial to determine latitudinal differences for modelling purposes, or the importance of priming and transgenerational priming for different origins of this species regarding its resilience towards climate change‐related stressors.

Our findings are opening the field of ecological epigenetics for marine brown algae. Given their sessile lifestyle, often low dispersal potential, and partial self‐fertilization, marine macroalgae are expected to benefit strongly from epigenetic variation and its potential for rapid acclimation and nongenetic adaptation. The potential for methylome‐based rapid evolution in these habitat‐forming ecosystem engineers is likely particularly important in the context of rapid ocean climate change as it may mitigate the predicted habitat loss in warm‐temperate regions, and facilitate invasion into the Arctic. Recognizing the functional role of the kelp methylome can be a first step to epigenetically optimize seedling and gametophyte propagation for sustainable management, restoration and cultivation, given the essential role of the epigenome for development. Furthermore, modifying the epigenome by tuning temperature rearing conditions may be exploited as a noninvasive bio‐engineering technique for strain improvement to ensure production security under environmental challenges that were not foreseen by breeding programmes.

## 6 GLOSSARY

**Gametophyte:** Microscopic life stage of kelp, divided into male and female individuals (1n).

**Sporophyte:** Macroscopic life stage of kelp (2n), which, e.g. constitute a kelp forest.

**Priming**: (artificial) exposure to a given stressor in order to create a stress memory to improve resilience during future encounters with the stressor.

**Transgenerational priming:** A stress memory which is heritable and can improve resilience across generations.

**Ecotype:** Genetically distinct variations of a species that are adapted to their respective environment Conner and Hartl (2004).

**Phenotype:** Capacity to react to local factors based on allelic differences.

**Phenotypic plasticity**: Individual, direct response of the phenotype to local factors within a single generation (Schlichting, 1986).

**Eco‐phenotype:** The combination and interaction of both genetic (DNA sequence‐related) and epigenetic mechanisms that lead up to a specific ‘adaptation’ of single individuals or origins to their respective environment, which can be passed on to following generations, and typically prevails for at least one generation when this generation encounters factors differing from those of the parental generation. With this, the definition of ‘adaptation’ would deviate from the classical one (‘heritable, but DNA sequence‐based’) towards ‘heritable, but transcription‐based’, as it would include epigenetic mechanisms influencing gene expression.

**Tags**: 32–33 bp sequences around in silico predicted methylation sites in CG or CHG sequence contexts.

**Reads**: DNA sequences obtained from the Ilumina sequencer.

**Mapped reads**: Reads that mapped back to any site within the target genome. One read can map back to several sites.

**Uniquely mapped reads**: Reads that only mapped back to one place in the target genome.

**Coverage**: Number of uniquely mapped reads across all samples for a certain site of mapping.

**Contig:** Assembly of sequenced reads of the same genetic origin.

**Origin**: Here, it is used in the latitudinal context of origin, of both lab and field samples. ‘Helgoland origin’ are those samples originating from 54°11′18.9″N 7°54′14.1″E, while ‘Spitsbergen origin’ are those originating from 78°59′26.0″N 11°58′42.3″E.

**Rearing condition**: In our study can be ‘wild’ (field samples) and ‘cultured’ (lab samples), with further distinction among ‘5’, ‘10’ and ‘15°C’ in the lab samples.

## CONFLICT OF INTEREST

No conflicts of interest were met.

## Supporting information

Supplementary Material

## Data Availability

Data for this study are available at NCBI SRA Bio Project PRJNA809008. Two data sets that were too big to store elsewhere are available at https://doi.org/10.6084/m9.figshare.19411460 (counts, Table S2) and https://doi.org/10.6084/m9.figshare.19411574 (reads per million, Table S3).
