# Supplementary material for: Differences by origin in methylome suggest eco‐phenotypes in the kelp Saccharina latissima
Source: Evol Appl. 2022 May 11;16(2):262–78. doi: 10.1111/eva.13382 (PMC9923482; doi:10.1111/eva.13382)
Supplement: Supplementary file 1 — Supplementary Material [file EVA-16-262-s001.zip › Supplementary caption.docx]

**Figure S1:**

Scheme of the beaker cultures, with four technical replicates (1-4) per origin (H = Helgoland, S = Spitsbergen) at the three temperatures. Each beaker contained several sporophytes. Of these, only one sporophyte each was randomly chosen as ‘the sample’ for the respective condition.

**Figure S2**

Annual water temperature measured by the underwater observatory (~ 54°11'N 7°54'E) at ~10m depth since 2018 in Helgoland. Compilation of temperature data obtained at the Pangea database (Fischer et al 2021 a, b, c).

**Figure S3**

Annual water temperature measured by the underwater observatory (78°59'49.61"N 11°58'08.55"E) at ~10m depth since 2018 in Ny-Ålesund, Svalbard. Compilation of temperature data obtained at the Pangea database (Fischer et al 2019, 2021 d, e).

**Figure S4**

GO terms enriched in the differentially methylated sites between samples of *Saccharina latissima* from Helgoland and Spitsbergen in sporophyte field samples (A) and lab cultures (B), shown for gene body (‘mRNA’) and regulatory regions (‘flanking’: 10 kbp upstream of genes to 200bp downstream of the annotated transcription start site, or 10kbp downstream of genes). Terms enriched in sites upregulated in the samples from Helgoland are shown with positive adjusted *p*-values, terms enriched in sites upregulated in samples form Spitsbergen with negative adjusted *p*-values. Adjustment according to Benjamini and Hochberg (1995). 1) BP: Biological Process, 2) CC: Cellular Component, 3) MF: Molecular Function. See Suppl. Table ST9

**Figure S5:**

GO terms enriched in the differentially methylated sites between sporophyte field samples and lab cultures of Saccharina latissima), shown for gene body (‘mRNA’) and regulatory regions (‘flanking’: 10 kbp upstream of genes to 200bp downstream of the annotated transcription start site, or 10kbp downstream of genes). Terms enriched in sites upregulated in the Field samples are shown with positive adjusted p-values, terms enriched in sites upregulated in Lab samples with negative adjusted p-values. Adjustment according to Benjamini and Hochberg (1995). BP: Biological Process, MF: Molecular Function. See Suppl. Table ST10

**Figure S6**

Assessment of microbiota in the sequenced DNA of one randomly chosen *S. latissima* lab sample A) in percentage of sequenced reads that could be assembled into contigs, and B) as a representation of taxa.

**Figure S7**

Overlap of differentially methylated sites between lab and field samples. Of the 375 observed differentially methylated sites, only five overlapped between lab and field samples. See Suppl. Table ST8

**Figure S8**

Overlap of differentially methylated sites between lab samples at 5 °C and 15 °C, Helgoland vs Spitsbergen. No differentially methylated sites were observed at 10 °C. The one site that was differentially methylated between Helgoland and Spitsbergen at 5 °C is one of the four sites observed at 15 °C. See Suppl. Table ST11

**Table ST1**

Data on mapping level. It shows how many of the produced reads mapped uniquely back to the genome of *S. japonica.*

**Table ST2**

Counts per sample and MethylRAD tag. The total number of rows gives the number of sites that are covered ≥3. The data is openly available at 10.6084/m9.figshare.19411460.

**Table ST3**

List of reads per million per sample and MethylRAD tag. The data is openly available at 10.6084/m9.figshare.19411574.

**Table ST4**

Mapping Overview (coverage) for lab and field samples; column C shows the number of methylated sites that could potentially be methylated, column E are those with a coverage of ≥3 per sample, column M gives the average coverage per sample, column N the mean coverage ± SD, column O the expected overall methylation level that would have been derived from applying a method that extracts all sequence contexts (like WGBS).

**Table ST5**

Statistics results for Fig.3; p-values are given in column G. Column B is used for grouping, and for statistical purposes all field samples were assigned the temperature value of ‘20’, even though real field temperatures deviated from this.

**Table ST6**

statistics results for Fig.4; p-values are given in column G. Column B is used for grouping, and for statistical purposes all field samples were assigned the temperature value of ‘20’, even though real field temperatures deviated from this.

**Table ST7**

Data of the differential expression analysis for Fig.5A

**Table ST8**

Data of the differential expression analysis for Fig.5B, and for Suppl. Fig. S7, Helgoland vs Spitsbergen Lab samples.

**Table ST9**

Data of the GO-Term analysis for Suppl. Fig. S4.

**Table ST10**

Data of the GO-Term analysis for Suppl. Fig. S5.

**Table ST11**

Results of the differential expression analysis for Suppl. Fig. S8, Helgoland vs Spitsbergen lab samples.
