# Supplementary figures and images for: Differences by origin in methylome suggest eco‐phenotypes in the kelp Saccharina latissima
Source: Evol Appl. 2022 May 11;16(2):262–78. doi: 10.1111/eva.13382 (PMC9923482; doi:10.1111/eva.13382)

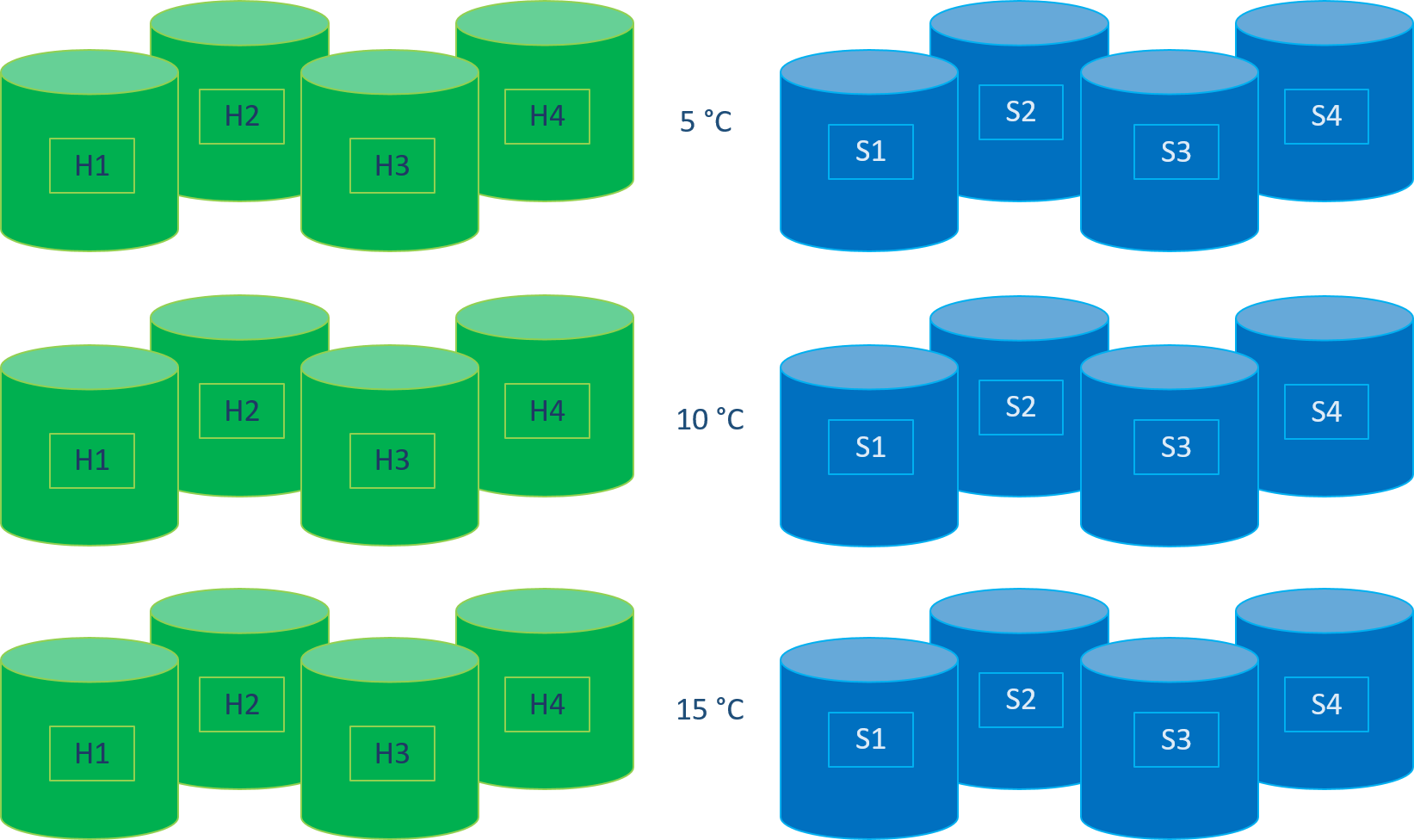

Supplement: Supplementary file 1 — Supplementary Material [file EVA-16-262-s001.zip › eva13382-sup-0001-FigS1.png]

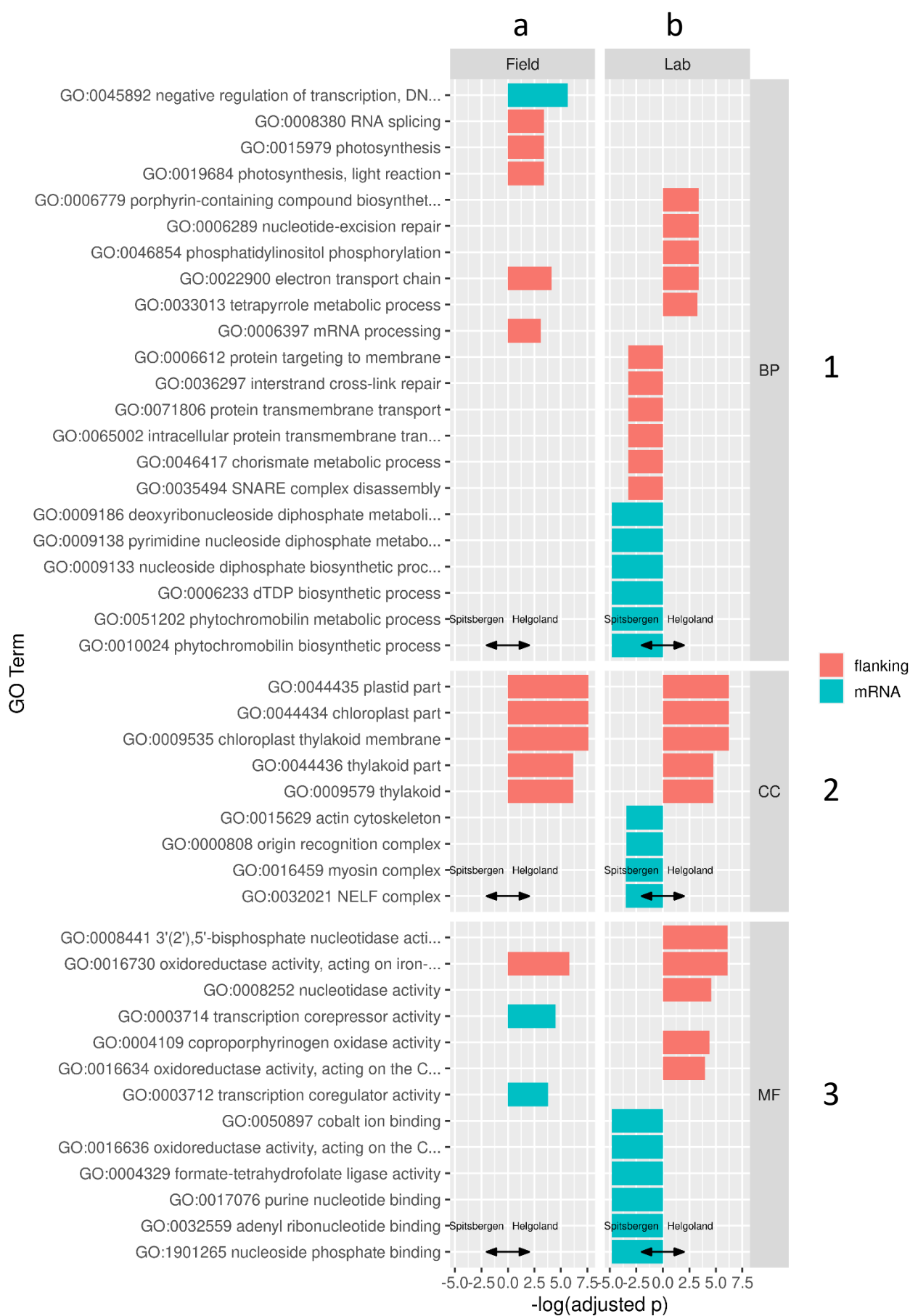

Supplement: Supplementary file 1 — Supplementary Material [file EVA-16-262-s001.zip › eva13382-sup-0001-FigS4.pdf]

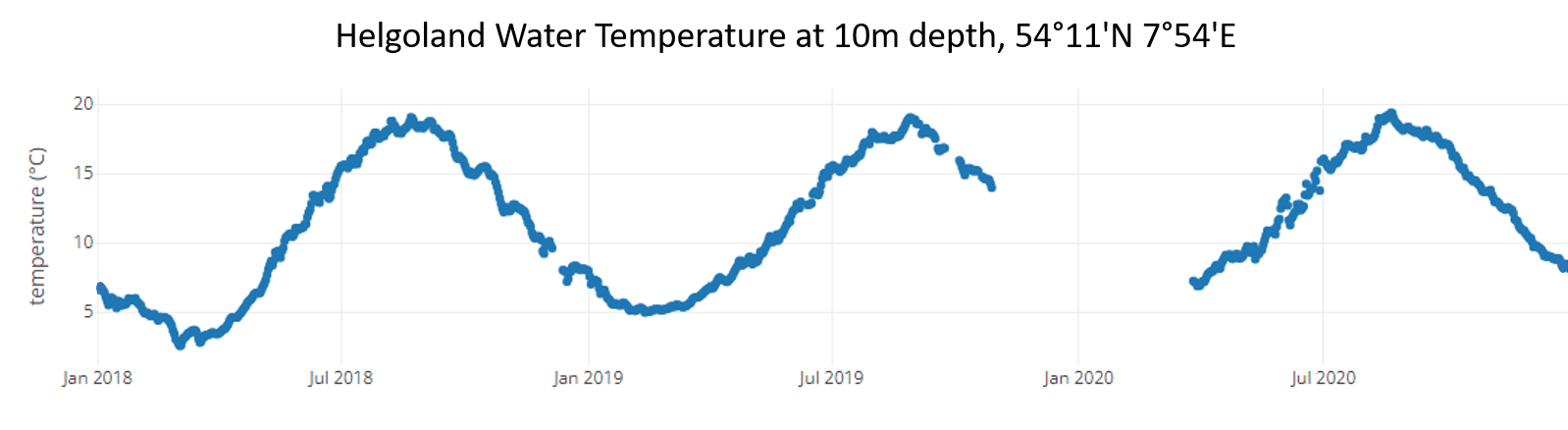

Supplement: Supplementary file 1 — Supplementary Material [file EVA-16-262-s001.zip › eva13382-sup-0002-FigS2.png]

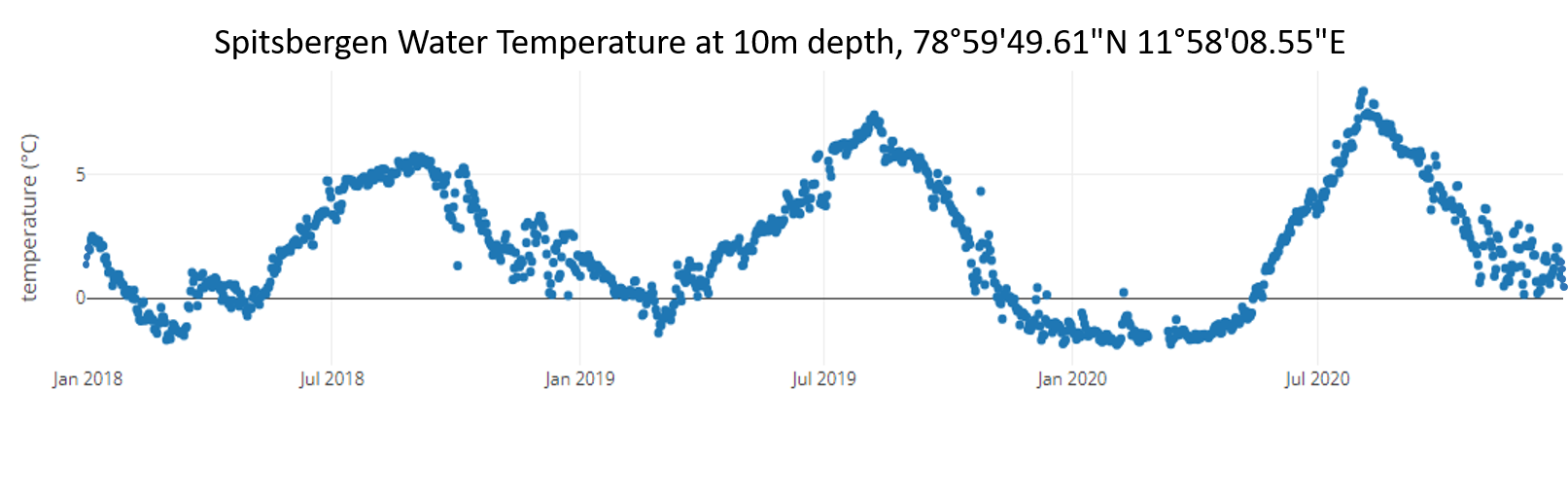

Supplement: Supplementary file 1 — Supplementary Material [file EVA-16-262-s001.zip › eva13382-sup-0003-FigS3.png]

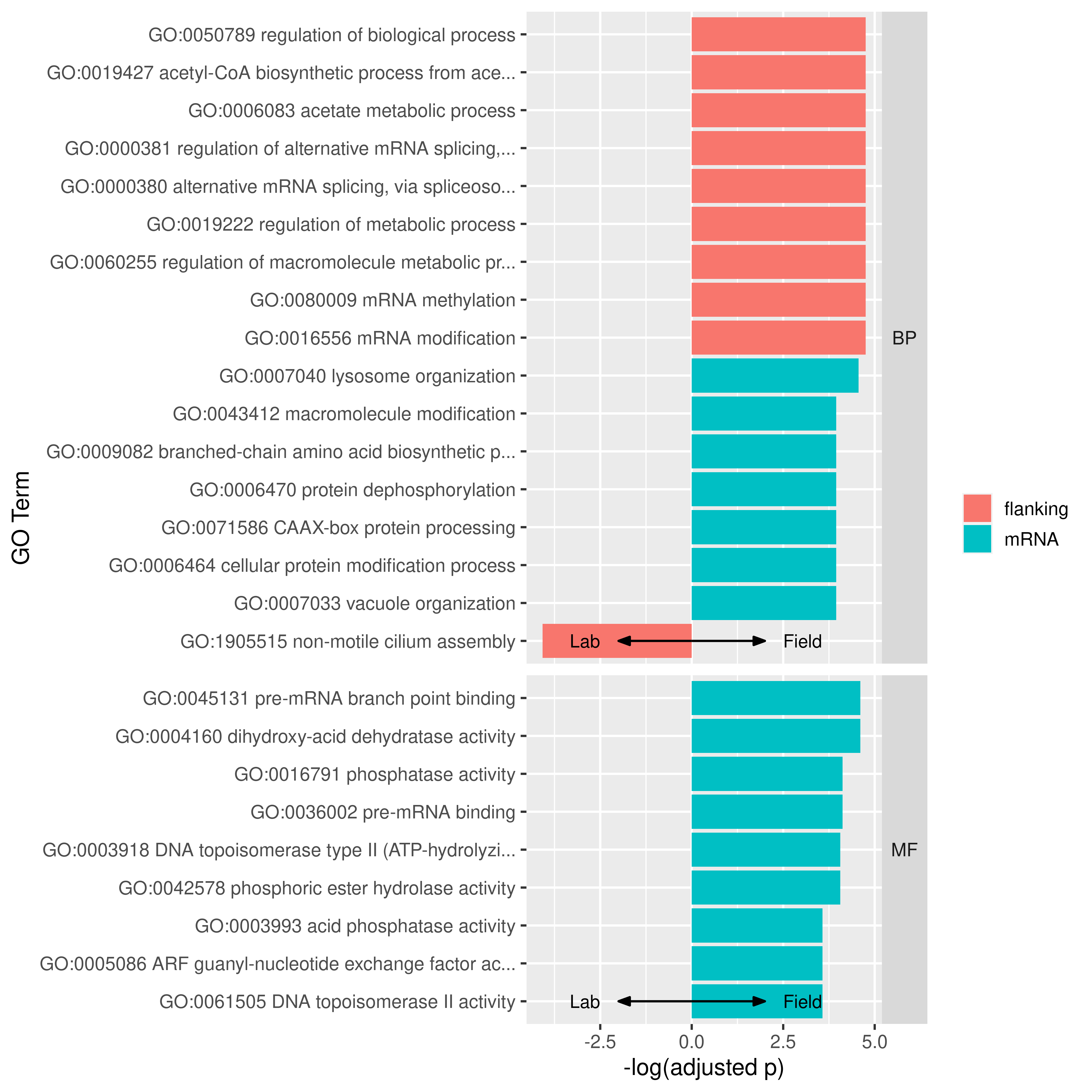

Supplement: Supplementary file 1 — Supplementary Material [file EVA-16-262-s001.zip › eva13382-sup-0005-FigS5.png]

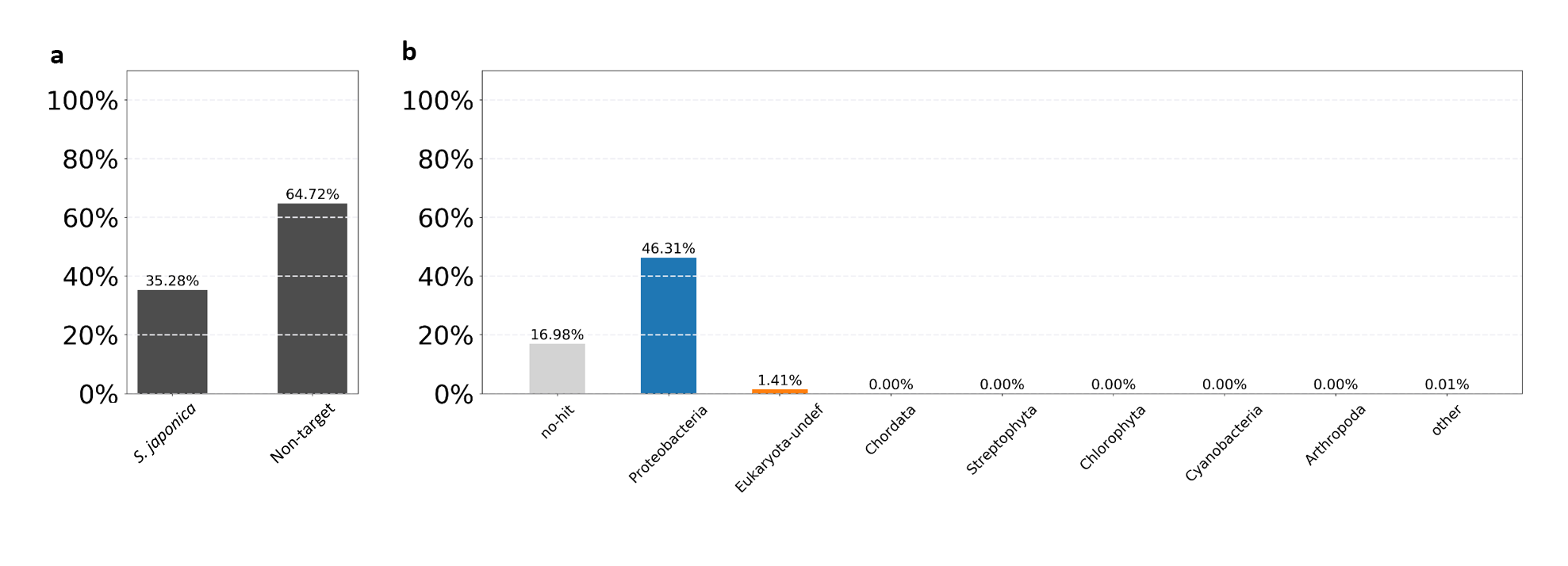

Supplement: Supplementary file 1 — Supplementary Material [file EVA-16-262-s001.zip › eva13382-sup-0006-FigS6.png]

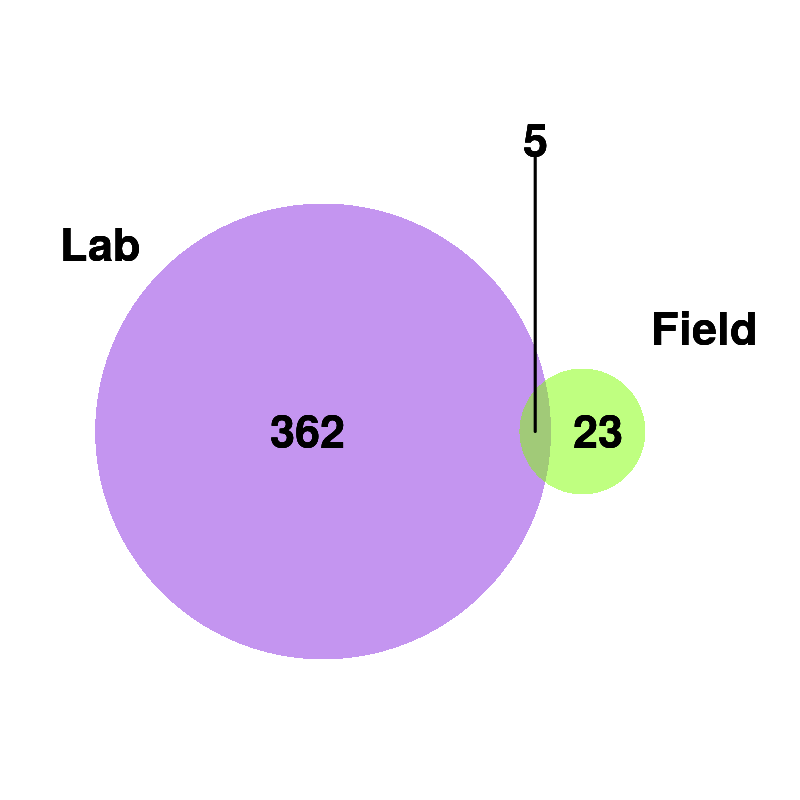

Supplement: Supplementary file 1 — Supplementary Material [file EVA-16-262-s001.zip › eva13382-sup-0007-FigS7.png]

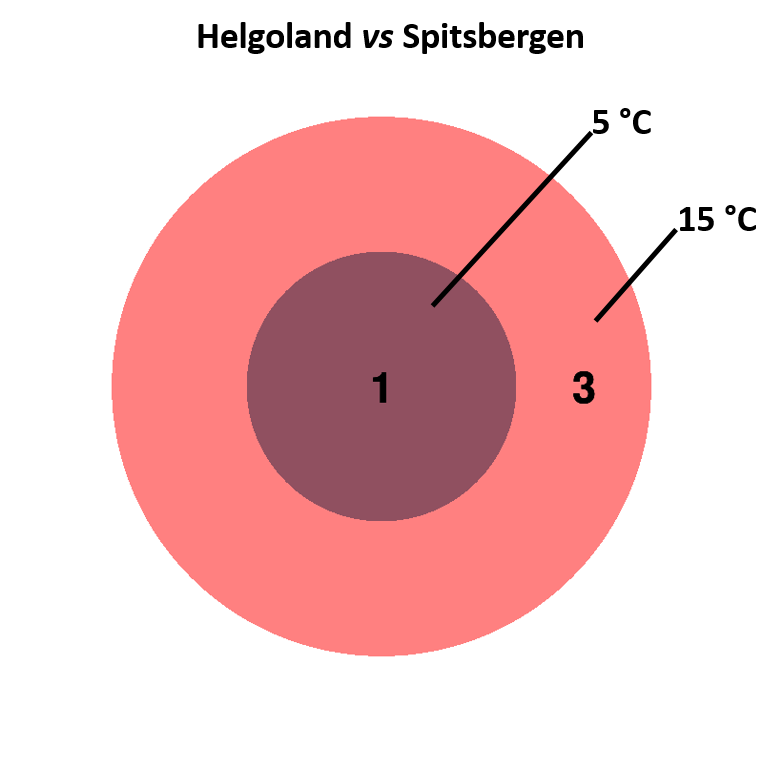

Supplement: Supplementary file 1 — Supplementary Material [file EVA-16-262-s001.zip › eva13382-sup-0008-FigS8.png]
